# Optimization of extracellular ethanol-tolerant β-glucosidase production from a newly isolated *Aspergillus* sp. DHE7 via solid state fermentation using jojoba meal as substrate: purification and biochemical characterization for biofuel preparation

**DOI:** 10.1186/s43141-021-00144-z

**Published:** 2021-03-24

**Authors:** Dina H. El-Ghonemy

**Affiliations:** Microbial Chemistry Department, Genetic Engineering and Biotechnology Research Division, National Research Centre, 33 El Buhouth St, Giza, 12622 Egypt

**Keywords:** *Aspergillus* sp. DHE7, Extracellular β-glucosidase, Purification, Biochemical characterization, Ethanol tolerance, Biofuel production

## Abstract

**Background:**

The increasing demand and the continuous depletion in fossil fuels have persuaded researchers to investigate new sources of renewable energy. Bioethanol produced from cellulose could be a cost-effective and a viable alternative to petroleum. It is worth note that β-glucosidase plays a key role in the hydrolysis of cellulose and therefore in the production of bioethanol. This study aims to investigate a simple and standardized method for maximization of extracellular β-glucosidase production from a novel fungal isolate under solid-state fermentation using agro-industrial residues as the sole source of carbon and nitrogen. Furthermore, purification and characterization of β-glucosidase were performed to determine the conditions under which the enzyme displayed the highest performance.

**Results:**

A fungus identified genetically as a new *Aspergillus* sp. DHE7 was found to exhibit the highest extracellular β-glucosidase production among the sixty fungal isolates tested. Optimization of culture conditions improved the enzyme biosynthesis by 2.1-fold (174.6 ± 5.8 U/g of dry substrate) when the fungus grown for 72 h at 35 °C on jojoba meal with 60% of initial substrate moisture, pH 6.0, and an inoculum size of 2.54 × 10^7^ spores/mL. The enzyme was purified to homogeneity through a multi-step purification process. The purified β-glucosidase is monomeric with a molecular mass of 135 kDa as revealed by the SDS-PAGE analysis. Optimum activity was observed at 60 °C and pH of 6.0, with a remarkable pH and thermal stability. The enzyme retained about 79% and 53% of its activity, after 1 h at 70 °C and 80 °C, respectively. The purified β-glucosidase hydrolysed a wide range of substrates but displaying its greater activity on *p*-nitrophenyl-β-D-glucopyranoside and cellobiose. The values of *K*_*m*_ and *V*_max_ on *p*-nitrophenyl β-D-glucopyranoside were 0.4 mM and 232.6 U/mL, respectively. Purified β-glucosidase displayed high catalytic activity (improved by 25%) in solutions contained ethanol up to 15%.

**Conclusion:**

β-glucosidase characteristics associated with its ability to hydrolyse cellobiose, underscore its utilization in improving the quality of food and beverages. In addition, taking into consideration that the final concentration of ethanol produced by the conventional methods is about 10%, suggests its use in ethanol-containing industrial processes and in the saccharification processes for bioethanol production.

**Graphical abstract:**

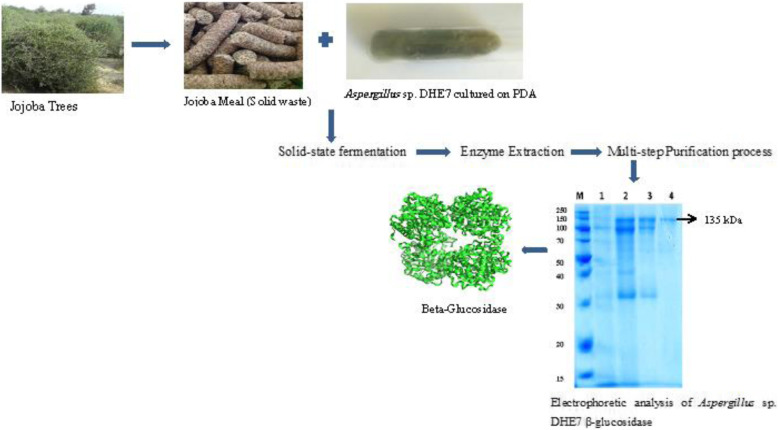

**Supplementary Information:**

The online version contains supplementary material available at 10.1186/s43141-021-00144-z.

## Background

The drastic depletion of fossil fuels associated with environmental problems has led to the search for new sources of biofuels and renewable energies. Cellulose is the main constituent of plant cell walls and the most abundant renewable carbon source in nature [1]. This, in turn, has created considerable interest in the conversion of agricultural biomass into fermentable sugars and therefore in the production of bioethanol. Thus, the production of bioethanol from lignocellulosic biomass is considered as one of the most important technologies for sustainable renewable transport fuels and may be a promising and potent biotechnological alternative [2]. However, the efficient degradation and hydrolysis of the cellulosic material to glucose requires the synergistic action of specific enzymes namely; endo-glucanases (E C 3.2.1.4) that internally hydrolyze the cellulosic chains and decrease in turn their polymerization level, exo-glucanases (E C 3.2.1.91), which attack non-reducing and reducing cellulose extremities, releasing cellobiose units from the molecule ends, and β-glucosidases (E C 3.2.1.21), a member of the glycosyl hydrolase groups, that hydrolyse cellobiose to glucose and other cello-dextrins, thus preventing its accumulation, which is considered as a strong inhibitor of endo-and exo-glucanases [3]. The ability of β-glucosidase to use various glycosidic substrates makes it appropriate for a variety of industrial processes, such as the enzymatic hydrolysis of cellulose for the production of fermentable sugars and the production of soy-derived functional foods. In the juice and beverage industry, the enzyme is often used where it can enhance the aromatic quality of wine as well as other grape derivative [4]. β-glucosidases are utilized also in a number of biotechnological processes, including the manufacture of new carbohydrate products, alcohol-based fuels, and the modification of flavonoids' shape and properties [5]. Moreover, they play an essential role in several biological processes, such as cyanide-based biodefense (CN released from cyano-glucoside) and the degradation of different harmful metabolites. In addition, β-glucosidases may be used in the synthesis of alkyl and aryl glycosides from natural polysaccharides and alcohols by reverse hydrolysis or trans-glycosylation, resulting in pharmaceutical products, detergent, and cosmetic industries [6].

β-glucosidases are ubiquitously distributed in all living kingdoms (mammals, plants and microorganisms); however, the microbial β-glucosidase is more advantageous than other sources of β-glucosidase due to its large scale fermentation production, low cost, free from any infectious viruses or prions, and environmentally friendly [7]. β-glucosidases often act as intracellular enzymes or remain bound to the cell wall in microorganisms. Hence, the lack of reports on extracellular β-glucosidases had encouraged the author to study the utilization of various agro-industrial residues as substrate for the production of this enzyme from a novel fungal source and under solid-state fermentation (SSF) conditions [8]. The iterative enhancement and advantages of using SSF have been reported in a number of studies, including the simplicity of growth conditions, reduced energy consumption, higher productivity rates, low catabolite repression, and increased stabilization of the produced enzymes. The use of agro-industrial waste as a substrate in SSF has received great interest because of the low-cost production and the reduction in environmental pollution resulted from the accumulation of these lignocellulosic residues [8].

The industrial applicability of β-glucosidase intimately depends on the expense of its production processes associated with choosing an appropriate agro-industrial substrate coupled with the selection of the hyper-producing strain and the optimization of different parameters utilized for growth and production of enzyme [9]. Among the various microbial sources, fungi were chosen for the current work because they are generally identified as the best cellulase producers and are preferably used in industrial applications owing to their large scale fermentation production, low cost, selectivity, specificity, and stability [9]. Hence, the objectives of this study were to produce an extracellular β-glucosidase from a new fungal isolate associated with low-cost fermentation method. Critical medium components have been optimized to achieve high β-glucosidase yields. Enzyme purification and biochemical characterization were investigated in order to determine the enzyme’s actions under different working conditions. In addition, the impact of ethanol, as a strong nucleophilic reagent, on enzyme activity was investigated since this enzyme is exposed to significant alcohol concentrations in several industrial applications.

## Methods

### Chemicals

*p*-Nitrophenyl-β-D-glucopyranoside (*p*-NPG), *p*-nitrophenol (*p*-NP), p-NP-β-D-galactopyranoside, pNP-α-D-glucopyranoside, p-NP-α-D-galactopyranoside, cellobiose, salicin, and bovine serum albumin (BSA) were purchased from Sigma Chemicals Company (St. Louis, MO, USA). DEAE-Sephadex A-50 and Sephadex G-100 were purchased from Pharmacia Fine Chemical (Sweden). Potato dextrose agar and broth (PDA and PDB) media and 3, 5-dinitrosalicylic acid (DNS) were purchased from Laboratorios Conda S.A., Madrid, Spain.

### Microorganism and inoculum preparation

Sixty filamentous fungi were isolated from various soil samples (Giza, Egypt) using serial dilution plate technique as follows: 1 gm of each soil sample was added to a clean flask containing 99 mL of sterile distilled H_2_O and the dilution was performed up to 10^−10^. Aliquots of 0.1 mL of soil sample suspensions were inoculated on Czapek Dox’s agar medium (g/L): glucose, 20; NaNO_3_, 2.0; KH_2_PO_4_, 1.0; KCl, 0.5; MgSO_4_.7H_2_0, 0.5; Rose Bengal, 0.05; agar, 20, adjusted at pH 5.0, and incubated at 28 °C for 4 days. Consequently, the developed distinct colonies were sub-cultured on PDA slants, incubated for 7 days at 28 °C, and finally stored at 4 °C. For inoculum preparation, the conidia of 7-day old PDA slant were gently scraped and 5 mL of a sterile saline solution containing 0.1% Tween 80 was added to each slant.

### Molecular identification and phylogenetic analysis

The pure culture mycelia of the selected fungal isolate (incubated at 28 °C for 2 days on PDB medium) were harvested by filtration, washed thoroughly with sterile distilled water and immersed in 1 mL of sterile Milli-Q H_2_O. The PrepMan Ultra (Applied Biosystem) and QiAamp DNA Mini Kit (Qiagen) were used to extract the genomic DNA from the fungal culture. A commercial kit; NEB, was used to conduct the polymerase chain reaction (PCR). Standard fungal primers; ITS1 5′-TCCGTAGGTGAACCTGCGG-3′ and ITS4 5′-TCCTCCGCTTATTGATATGC-3′ were used for amplification. The PCR amplification reaction, which consists of: 1 μL of DNA, 12.5 μL 2 Taq PCR Master Mix (contain 0.5 U Taq DNA polymerase/μL, 500 μM of each dNTP, Tris-HCl (20 mM, pH 8.3), 100 mM of KCl, 3 mM of MgCl_2_, bromophenol blue, 1 μL of each primer (10 μM) and 9.5 μL double-distilled H_2_O, was performed according to Oetari et al. [10] as follows: initial denaturation at 95 °C for 3 min, followed by 35 denaturation cycles at 95 °C for 15 s, annealing at 58 °C for 15 s, extension at 72 °C for 30 s, and final extension at 72 °C for 2 min.

The products of PCR were analyzed by electrophoresis using 1% (w/v) agarose gel, stained for 25 min with ethidium bromide of 0.04% (v/v), and finally identified under the light of UV. Sequencing was carried out by Macrogen Company, Seoul, South Korea. Basic local alignment search tools (BLAST) had been used to find homology with the sequences of known fungal sp. [11]. All amplified sequences of the ITS regions have been deposited in GenBank and compared with other sequences by employing the BLASTN program (http://blast.ncbi.nlm.nih.gov/Blast.cgi) and aligned with similar sequences by using CLUSTX. Phylogenetic tree was assembled using a neighbor-joining approach by employing MAGA 4.1 program [12].

### Extracellular β-glucosidase production by different fungal isolates under solid-state fermentation

Wheat bran was used as the basic substrate and the fermentation process was performed as follows: 1 mL aliquot of fungal spore suspension of 1.5 × 10^7^ spores/mL was transferred to Erlenmeyer flask (500 mL) containing 10 g of wheat bran moistened (up to 60%) with the basal medium of the following composition (g/L): KH_2_PO_4,_ 2.0; MgSO_4_.7H_2_O, 0.5; KCl, 0.5; adjusted at pH 5.5. The inoculated flasks were incubated for 96 h under static fermentation conditions at 28 °C.

### Enzyme extraction

The extraction of the crude enzyme from the fermented substrate was performed by adding 100 mL of sterile distilled water followed by shaking on a rotary shaker (180 rpm) for 60 min at room temperature. The mixture developed was squeezed using a muslin cloth, filtered through Whatman no. 1 filter paper, and centrifuged at 5000×*g* for 10 min at 4 °C. The clear supernatant was utilized as a crude-enzyme extract for the determination of extracellular β-glucosidase activity.

### Enzyme assay and estimation of protein

The β-glucosidase activity was assayed by measuring *p-*NPG hydrolysis according to the method described by Cai et al. [13]. Briefly, in a clean test tube, 100 μL of properly diluted enzyme solution was thoroughly mixed with 400 μL of 5 mM *p-*NPG in 0.05 M Na-citrate buffer (pH 5.0). The reaction mixture was incubated for 10 min at 50 °C, and then 1 mL of 0.5 M ice-cold Na_2_CO_3_ was added to terminate the reaction. The amount of *p-*nitrophenol released was quantified at 410 nm in Cary-100 UV-Vis–Spectrophotometer (Agilent Technologies, Germany). One unit (U) of β-glucosidase activity was defined as the amount of enzyme that released 1 *μ*mol of *p-*NP per minute under the standard assay conditions. Enzyme yield was expressed as β-glucosidase activity per gram on dry mass substrate (U/g ds). The protein content was evaluated according to Bradford method [14] and BSA was used as a standard. Specific enzyme activity was expressed as unit/milligram protein (U/mg).

### Optimization of different physico-chemical parameters for β-glucosidase production under SSF

The selected fungal isolate was cultivated in Erlenmeyer flasks (500 mL) containing 10 g of each substrate (wheat bran, wheat germ, cotton seed waste, sugarcane bagasse, sugar beet pulp, sunflower oil cake, jojoba meal, rice bran, rice straw, and rice husk; all substrates were washed thoroughly with distilled water and dried at 65 °C), moistened with the basal medium described above up to a moisture level of 60% (w/v). The selected substrate was further used in the subsequent studies to investigate the impacts of varying the pH value of the production medium (from 4.0 to 8.0 using 0.5 M HCl/NaOH), moisture content (from 33 to 90%), inoculum size (from 0.64 × 10^7^ to 5.08 × 10^7^), incubation temperature (from 25 to 45 °C), and time course (at 24 h interval) on the production of enzyme by the selected fungal strain. The optimal parameter in each step was further used in an iterative strategy performed to optimize the cultivation process for maximum β-glucosidase production.

### Enzyme purification

All purification steps were performed at 5 *°*C. The crude-enzyme extract was precipitated by the gradual addition of cold ethanol (− 20 °C) with gentle stirring to give up to 30% saturation, and stand for 24 h at 5 °C followed by centrifugation at 10,000*×g* for 15 min at 4 °C. The pellet was removed and the supernatant was further saturated with cold ethanol up to 60% and maintained overnight at 4 °C followed by centrifugation at 5 °C for 10 min at 10,000*×g*. The developed pellet was dissolved in a minimal volume of 0.05 M Na-citrate (pH 5.0) and dialyzed overnight towards the same buffer at 4 °C. The dialysate was loaded onto DEAE-sephadex A-50 column (1.5 × 45 cm) pre-equilibrated with 0.05 M Na-citrate buffer (pH 5.0). The adsorbed protein has been washed twice the bed volume using the same buffer. A linear gradient of NaCl ranging from 0.1 to 1.0 M in 0.05 M Na-citrate buffer (pH 5.0) was used for protein elution at a flow rate adjusted to 20 mL/h. Fractions of 5 mL were collected and analyzed for β-glucosidase activity and protein content. Active fractions with maximum activity of β-glucosidase were pooled together, dialyzed against the above buffer, and concentrated by lyophilization (− 50 °C) for the next purification step. The concentrated sample was added to a Sephadex G-100 column (1.5 × 50 cm) pre-equilibrated with Na-citrate buffer (0.05 M, pH 5). Protein elution was run at a flow rate of 25 mL/h using the same buffer. Fractions with high β-glucosidase activity were collected, dialyzed against the same buffer, lyophilized, and checked for pureness using sodium dodecyl sulphate polyacrylamide gel electrophoresis (SDS-PAGE).

### Sodium dodecyl sulfate polyacrylamide gel electrophoresis analysis

The molecular mass of the purified β-glucosidase was calculated by the SDS-PAGE (Bio-Rad) technique as described by Laemmli [15], using 0.1% SDS in a 12.5% separating gel (pH 8.8) and 5.0% stacking gel (pH 6.8). After running, the gel was stained with R-250 Coomassie Brilliant Blue for 12 h under shaking (80 rpm) and decolorized twice in methanol-acetic acid solution. The bands formed were visualized, photographed while being wet, and dried. The molecular mass was determined by calibration against the standard protein markers (Fermentas, spectra ^TM^ wide range protein ladder ranging from 15 to 250 kDa).

### Biochemical characterization of the purified *Aspergillus* sp. DHE7 β-glucosidase

#### Impact of temperature and pH

The optimal temperature for β-glucosidase activity was determined by carrying out the reaction at various temperatures between 30 and 90 °C, with an increment of 5 °C at the standard assay procedure. Thermal stability was evaluated by incubating the enzyme at the desired temperature (from 30 °C to 90 °C) for different time intervals. The non-incubated enzyme was used as the control (100%). The impact of pH on the purified β-glucosidase activity was studied by measuring the activity at the optimum temperature at various pH values ranging from 3.0 to 9.0 (using 0.05 M of various buffer systems namely Na-citrate, pH 3–6; citrate-phosphate, pH 6–7; Tris-HCl, pH 7–9). The pH stability of β-glucosidase was evaluated by incubating the enzyme (without the substrate) in the above-mentioned buffers for 24 h at 5 °C and measuring the residual activity as previously described. The non-treated enzyme activity was regarded as control (100%).

#### Influence of different additives on β-glucosidase activity

The effects of various metal ions (Fe^2+^, Pb^2+^, K^+^, Ca^2+^, Mg^2+^, Na^+^, Cu^2+^, Zn^2+^, Co^+^, Mn^+^, and Hg^2+^) and chemical reagents (EDTA, DMSO, glycerol, and β-mercaptoethanol (β-ME) ) on the activity of *Aspergillus* sp. DHE7 β-glucosidase were tested by incubating the corresponding additive at a final concentration of 5 mM with the purified enzyme at 25 °C for 1 h. The residual activity was measured as described above using p-NPG as substrate. The enzyme activity without an additive was regarded as a control (100%).

#### Substrate specificity

The enzyme substrate specificity was studied against various substrates. Identical reaction mixtures containing equal amount of enzyme solution were prepared; each received an equimolar amount of the *p*-NP (i.e., *p*-NPG, *p*-NP-α-D-glucopyranoside, *p*-NP-β-D-galactopyranoside, and *p*-NP-α-D-galactopyranoside), disaccharide (i.e., cellobiose, salicin, lactose, maltose, sucrose), and polysaccharide (i.e., CMC, starch) substrates. For *p*-NP substrates, all reaction mixtures were incubated at the optimal temperature for 10 min and the released *p*-NP was determined as previously described. In the case of other substrates, the released reducing sugars (expressed as glucose equivalent) were determined using the DNS method (A_540_) [16]. One unit of β-glucosidase activity was defined as the amount of enzyme that released one μmol of *p*-NP or D-glucose equivalents/min under the standard assay conditions.

#### Kinetic parameters

Maximum velocity (*V*_max_) and Michaelis–Menten constant (*K*_*m*_) of the purified *Aspergillus* sp. DHE7 β-glucosidase were calculated using various concentrations (0.25 to 20 mM) of *p-*NPG as a substrate according to the nonlinear regression method of Lineweaver and Burk [17]. The reactions were carried out under the optimal assay conditions.

### Influence of various concentrations of ethanol on *Aspergillus* sp. DHE7 β-glucosidase activity

Impact of ethanol as a strong nucleophilic reagent on β-glucosidase activity was evaluated by adding the organic solvent at different concentrations (0–40%) to the reaction mixture containing 20 μg of β-glucosidase and 5 mM *p*-NPG (dissolved in 0.05 M Na-citrate buffer, pH 6.0). All the reaction mixtures were incubated under the optimal assay conditions of pH and temperature. The residual enzyme activities were measured as previously described. The non-treated enzyme activity was used as a control (100%).

### Statistical analysis

All experiments were carried out in triplicate. Data were collected, revised, and entered to the statistical package for social science (SPSS) version 23; the quantitative parametric data were presented as mean and standard deviation (SD).

## Results

### Screening of various fungal isolates for the production of extracellular β-glucosidase

Sixty isolated filamentous fungi were screened for their abilities to produce extracellular β-glucosidases under SSF after 96 h using wheat bran as a substrate. Results shown in Table 1 revealed that 38 fungal isolates out of the tested set produced extracellular β-glucosidases in various proportions; however, the highest production was exhibited by the isolate DHE7 (81.3 ± 4.2 U/g ds) followed by isolate DHE20 (65.9 ± 3.4 U/g ds) and isolate DHE33 (58.8 ± 2.7 U/g ds). Therefore, the isolate DHE7 was selected for the subsequent identification studies.

**Table 1:** Screening of different fungal isolates for extracellular β-glucosidase (β-GL) production (on dry mass) under solid state fermentation using wheat bran as substrate

Microorganism	β-GL activity/(U/g)	Microorganism	β-GL activity/(U/g)	Microorganism	β-GL activity/(U/g)
DHE1	35.2 ± 2.4	DHE21	8.6 ± 0.4	DHE41	6.4 ± 0.3
DHE2	00.00	DHE22	00.00	DHE42	00.00
DHE3	22.3 ± 1.9	DHE23	00.00	DHE43	00.00
DHE4	22.7 ± 1.3	DHE24	11.4 ± 0.2	DHE44	8.7 ± 0.2
DHE5	00.00	DHE25	00.00	DHE45	00.00
DHE6	29.3 ± 2.1	DHE26	7.5 ± 0.1	DHE46	25.5 ± 1.3
DHE7	81.3 ± 4.2	DHE27	10.3 ± 0.4	DHE47	37.2 ± 1.7
DHE8	34.6 ± 1.2	DHE28	42.7 ± 1.1	DHE48	00.00
DHE9	45.5 ± 2.6	DHE29	17.2 ± 1.4	DHE49	00.00
DHE10	00.00	DHE30	00.00	DHE50	17.6 ± 1.2
DHE11	20.2 ± 0.5	DHE31	00.00	DHE51	10.4 ± 0.3
DHE12	10.3 ± 0.2	DHE32	8.9 ± 0.1	DHE52	9.2 ± 0.2
DHE13	00.00	DHE33	58.8 ± 2.7	DHE53	00.00
DHE14	12.7 ± 0.3	DHE34	00.00	DHE54	00.00
DHE15	11.1 ± 0.4	DHE35	7.7 ± 0.1	DHE55	6.4 ± 0.1
DHE16	00.00	DHE36	5.6 ± 0.2	DHE56	24.3 ± 1.3
DHE17	24.3 ± 1.1	DHE37	00.00	DHE57	9.6 ± 0.7
DHE18	28.2 ± 1.3	DHE38	00.00	DHE58	6.5 ± 0.2
DHE19	00.00	DHE39	9.8 ± 0.1	DHE59	00.00
DHE20	65.9 ± 3.4	DHE40	12.3 ± 0.2	DHE60	8.7 ± 0.3

Data are expressed as mean value ± S.D. of triplicate measurements

### Molecular identification

**Fig. 1:**
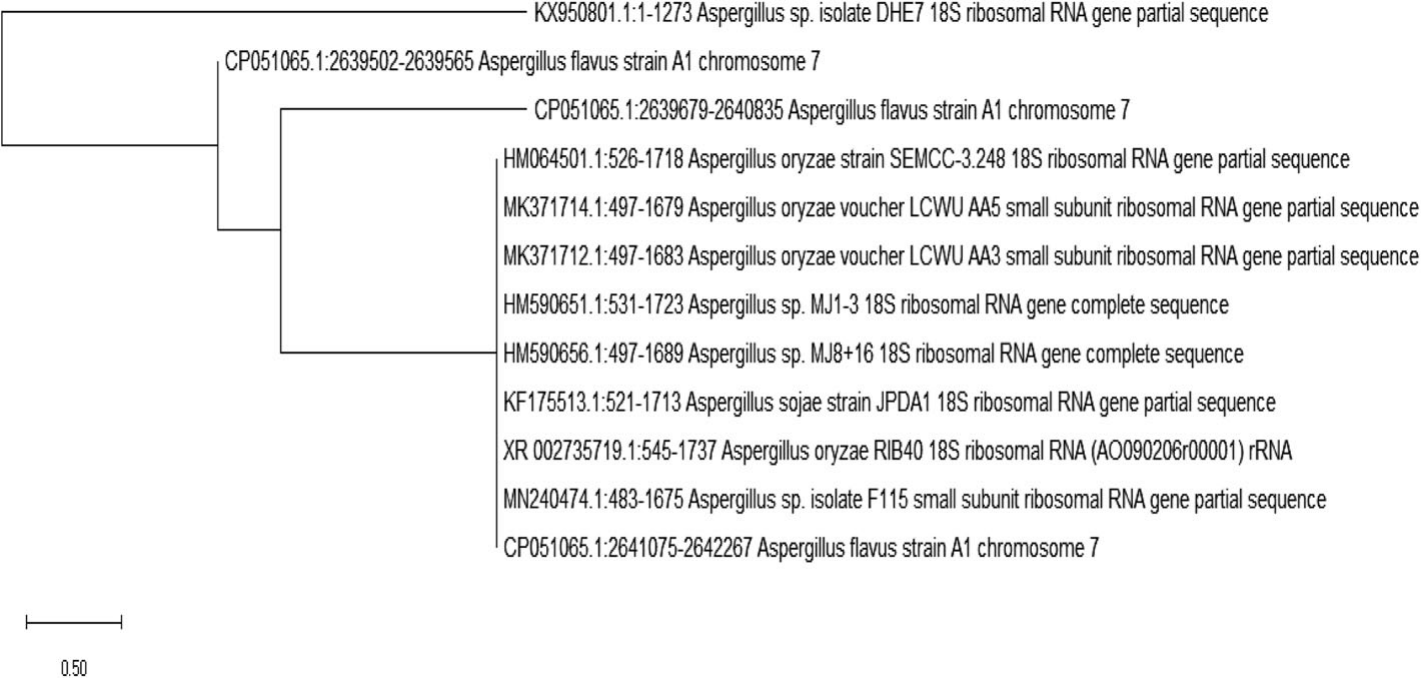
Phylogenetic tree of *Aspergillus* sp. DHE7 based on the results of polymerase chain reaction (PCR) amplification

The PCR results revealed an efficient amplification (**S.** **1**); with a single band of 1.3 kbp amplified DNA (**S.** **2**). The fungal isolate DHE7 was genetically identified as a new *Aspergillus* sp. DHE7 under the accession number of KX950801. The amplified nucleotide sequences were submitted to GenBank via the NCBI and compared with the most closely related *Aspergillus* species and their percentages of identity (**S.** **3**) (http://www.ncbi.nlm.nih.gov/Banklt). The phylogenetic tree (Fig. 1) and multiple-sequence alignment of the sequences of amplified targeted ITS region were constructed using the CLUSTALW (http://align.genome.jp) procedure (**S.** **4**).

### Optimization of various physiological conditions for maximum β-glucosidase production under SSF

#### Selection of the agro-industrial substrate

Among the agro-industrial substrates tested, the cultivation of *Aspergillus* sp. DHE7 on jojoba meal containing medium yielded a higher level of β-glucosidase of 116.4 ± 2.4 U/g ds, followed by wheat bran medium, 84.3 ± 4.4 U/g ds, and cotton seed waste medium, 78.5 ± 2.5 U/g ds, while a negligible amount of enzyme activity was detected in rice husk and rice straw media (Table 2). Subsequently, jojoba meal was selected as the best substrate for further studies in order to optimize the culture conditions for maximum extracellular β-glucosidase production from *Aspergillus* sp. DHE7.

**Table 2:** Effect of various physicochemical parameters on extracellular β-glucosidase (β-GL) production (on dry mass basis) by *Aspergillus* sp. DHE7 in solid state fermentation (SSF)

Agro-industrial substrate	β-GL activity/(U/g)
Jojoba meal	116.4 ± 2.4
Rice husk	13.8 ± 0.8
Rice straw	15.1 ± 0.3
Sugar beet bulb	32.9 ± 1.2
Sugarcane bagasse	27.3 ± 1.9
Sunflower oil cake	43.8 ± 1.4
Cotton seed waste	78.5 ± 2.5
Olive oil cake	23.1 ± 0.4
Rice bran	33.2 ± 1.1
Wheat bran	84.3 ± 4.4
Wheat germ	54.6 ± 2.1
pH	
4.0	44.3 ± 2.4
5.0	117.3 ± 4.1
6.0	126.4 ± 3.7
7.0	104.5 ± 3.6
8.0	86.5 ± 2.1
Moisture/%	
33	56.2 ± 1.4
50	82.4 ± 2.6
60	125.7 ± 2.8
70	141.6 ± 4.6
75	124.5 ± 3.2
80	89.3 ± 2.8
85	77.5 ± 1.7
90	63.2 ± 2.3
Incubation/h	
24	00.00
48	55.1 ± 1.5
72	152.8 ± 4.3
96	139.4 ± 2.6
120	119.7 ± 2.4
Temperature/C°	
25	93.1 ± 2.3
30	151.2 ± 3.4
35	167.2 ± 5.1
40	112.6 ± 4.2
45	77.5 ± 3.7
Inoculum size/(spore/mL)	
0.64 × 10^7^	96.2 ± 3.1
1.27 × 10^7^	163.5 ± 4.9
2.54 × 10^7^	174.6 ± 5.8
3.81 × 10^7^	143.2 ± 4.3
5.04 × 10^7^	106.1 ± 2.8

Data are expressed as mean value ± S.D. of triplicate measurements

#### Effect of different fermentative parameters on β-glucosidase production using jojoba meal as substrate

The production of β-glucosidase by the selected fungus was increased steadily with the increase in the pH value reaching its maximum at an initial pH of 6.0 (126.4 ± 3.7 U/g ds) after 96 h of growth at 30 °C (Table 2). In addition, the fungus displayed a considerable enzyme production for all pH values tested. However, when increasing the initial pH of the production medium above the optimum value of 6.0, a slight decline in enzyme production was observed. On the other hand, the lowest enzyme yield of 44.3 ± 2.4 U/g ds was detected at lower initial medium pH of 4.0. Among the moisture levels tested, maximal enzyme yield of 141.6 ± 4.6 U/g ds was observed in jojoba meal medium with an initial moisture content of 70% after 96 h of incubation at 30 °C and initial medium pH of 6.0 (Table 2). Different time intervals ranging from 24 to 120 h were tested to determine the optimum incubation period for maximum β-glucosidase production from the selected fungus. Results shown in Table 2 revealed that the highest β-glucosidase level of 152.8 ± 4.3 U/g ds was achieved after 72 h of incubation using jojoba meal as a substrate with an initial moisture level of 70% and pH value of 6.0. On the other hand, a decline in enzyme production was detected with the further increase in the incubation period.

Higher yields of β-glucosidase were produced by *Aspergillus* sp. DHE7 when grown at temperatures between 30 °C and 40 °C, while the optimum β-glucosidase yield of 167.2 ± 5.1 U/g ds was observed at 35 °C after 72 h of incubation (Table 2). Moreover, a decrease in enzyme production was observed in cultures incubated at temperatures above or below the optimal temperature 35 °C with least activity detected at incubation temperature of 45 °C (Table 2). Maximum β-glucosidase production of 174.6 ± 5.8 U/g ds by *Aspergillus* sp. DHE7 was occurred with an inoculum size of 2.54 × 10^7^ spores/mL after 72 h of incubation at 35 °C and pH of 6 (Table 2). However, a negative impact on enzyme production has been observed at inoculum levels higher or smaller than the optimum level.

### Purification of β-glucosidase from *Aspergillus* sp. DHE7

**Fig. 2:**
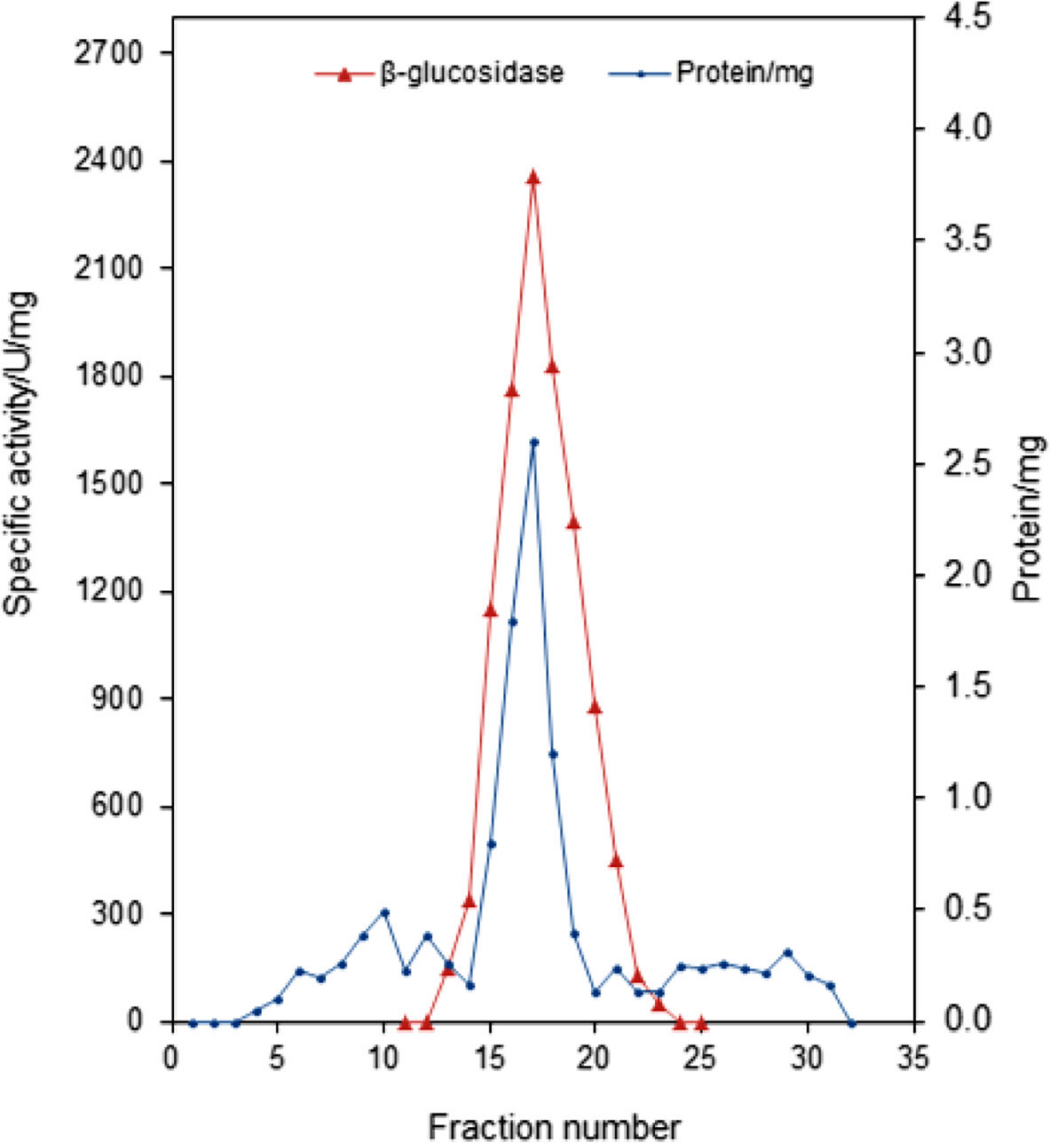
Elution diagram of β-glucosidase of *Aspergillus* sp. DHE7 from Sephadex G-100 column

**Fig. 3:**
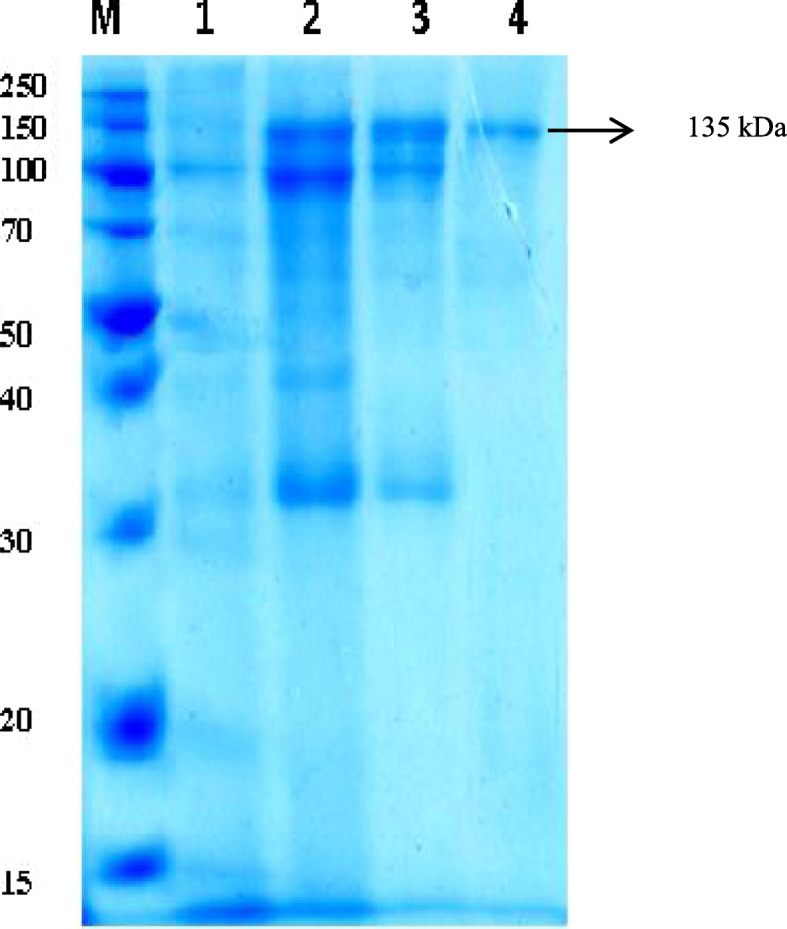
Electrophoretic analysis of an extracellular *Aspergillus* sp. DHE7 β-glucosidase. Separation was performed on a 12.5% (w/v) SDS Polyacrylamide gel and stained with Coomassie brilliant blue. From *left* to *right*: *M* standard protein markers, *lane 1* crude-enzyme extracts, *lane 2* fractional precipitation by chilled ethanol, *lane 3* partial purified β-glucosidase on DEAE-Sephadex A-50, *lane 4* purified β-glucosidase on Sephadex G-100

The crude culture filtrate obtained under the optimal fermentation conditions was used in the trials of getting a purified β-glucosidase and data reported were summarized in Table 3. The enzyme was purified 29.6-fold with a final recovery of 45.2%. The total amount of protein was decreased from 678 ± 5.7 to 6.3 ± 0.1 mg, while the specific activity was greatly increased from 91.3 to 2338.3 U/mg. The purified *Aspergillus* sp. DHE7 β-glucosidase was elucidated as a single active peak during gel filtration chromatography (Fig. 2), and its purity was confirmed by the existence of a single band with a molecular mass of approximately 135 kDa after staining on a 12.5% SDS-PAGE gel (Fig. 3).

**Table 3:** Summary of purification profile of *Aspergillus* sp. DHE7 β-glucosidase

Purification step	Total activity U	Total protein (mg)	Specific activity (U/mg)	Recovery (%)	Purification fold
Crude enzyme extract	43653 ± 153	678 ± 5.7	91.3	100	1.0
Ethanol precipitation	40685 ± 132	246 ± 3.8	165.4	93.2	1.8
Dialysis	36974 ± 145	213 ± 2.1	173.6	84.7	1.9
DEAE-Sephadex A-50	28505 ± 105	22.3 ± 0.8	1278.3	65.3	14.0
Sephadex G-100	14731 ± 127	3.8 ± 0.1	3876.6	33.7	42.5

Data are expressed as mean value ± S.D. of triplicate measurements

### Biochemical characterization of the purified *Aspergillus* sp. DHE7 β-glucosidase

#### Impact of temperature and pH on enzyme activity and stability

**Fig. 4:**
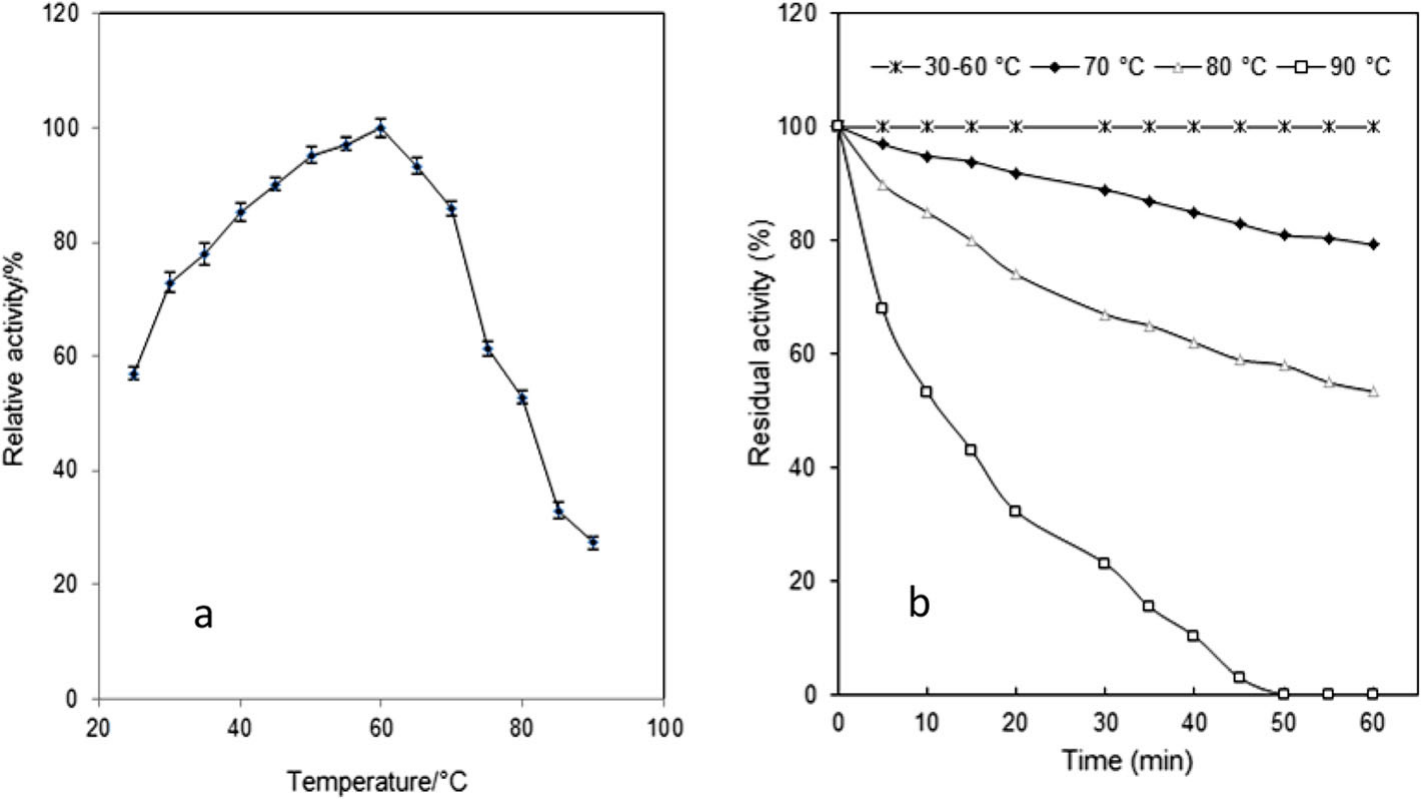
Effect of temperature on activity and stability of *Aspergillus* sp. DHE7 β-glucosidase: **a** Effect of temperature was determined by assaying the enzyme at different temperatures ranging from 25 to 90 °C, at pH 5.0 (0.05 M Na-citrate) against *p*-NPG as substrate. **b** Thermal stability behavior of *Aspergillus* sp. DHE7 β-glucosidase was evaluated by pre-incubating of the purified enzyme at different temperatures (pH 6.0, 30–90 °C) in the absence of substrate and assayed for residual activity on *p*-NPG under standard assay conditions

The impact of temperature on β-glucosidase activity was investigated under the standard assay conditions. According to Fig. 4a, it was found that the optimum temperature for β-glucosidase activity was 60 °C, while a gradual reduction in enzyme activity was observed at higher or lower temperatures than the optimum temperature. Regarding thermal stability studies, no significant loss in β-glucosidase activity was observed after 1 h of incubation at temperatures ranging from 25 to 60 °C (Fig. 4b). Furthermore, the enzyme retained about 79.3% and 53.4% of its initial activity, by increasing the temperature up to 70 °C and 80 °C, respectively. While at 90 °C, the enzyme retained about 34% and 23% of its activity, after 15 and 30 min of incubation, respectively.

**Fig. 5:**
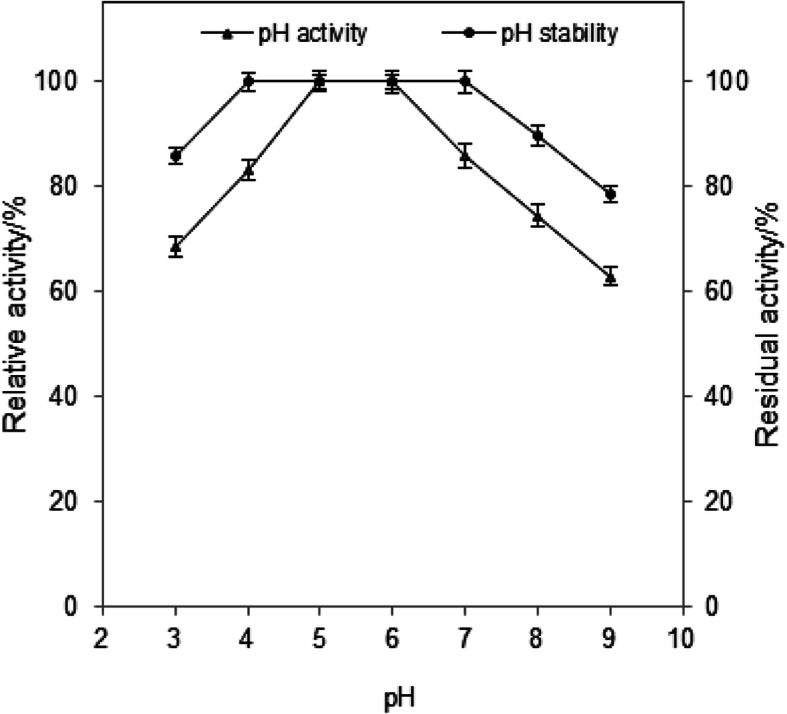
Effect of pH on *Aspergillus* sp. DHE7 β-glucosidase activity. Optimal pH for β-glucosidase activity (filled triangles) was determined by assaying the enzyme in various pH ranging from 3.0 to 9.0, pH stability (filled circles) was determined by pre-incubating of the purified β-glucosidase (without the substrate) in the same buffers for 24 h at 4 °C and measuring the residual activity. The non-treated enzyme activity was regarded as 100%

Optimum pH of the purified β-glucosidase activity was observed at pH value of 6.0 using 0.05 M Na-citrate buffer (Fig. 5), which was higher than those investigated by many authors for fungal β-glucosidases. However, a decline in β-glucosidase activity was observed at pH values below or higher than the optimum pH. The purified enzyme displayed remarkable pH stability, as it maintained its activity after 24 h of incubation at pH values between 4 and 7 at 5 °C, while maintained about 86.8% and 78.6% of its initial activity at pH 8 and 9, respectively (Fig. 5). It is worth note that the enzyme stability did not vary substantially at acidic pH, as it retained 85.9% of its activity at pH 3.0 after 24 h of incubation at 5 °C.

#### Effect of different additives

Among different metal ions and chemical reagents examined, a significant increase in enzyme activity was reported with Zn^2+^ (145%), K^+^ (137%), Mg^2+^ (129%) and SH-modifying β-mercaptoethanol reagent (149%), while a slight increase in enzyme activity was observed with Na^+^ (111%), Ca^2+^ (108%) and Fe^2+^ (107%) when used at a final concentration of 5 mM as shown in Table 4. In contrast, the addition of Mn^+^, Pb^2+^, Hg^2+^, Cu^2^, and Co^+^ decreased enzyme activity by 11%, 15%, 29%, 30%, and 35%, respectively. The thiol group inhibitor Ag^+^ had strongly inhibited B-glucosidase activity (Table 4). EDTA, a metal chelating agent, did not inhibit β-glucosidase activity. On the other hand, DMSO stimulated β-glucosidase activity by 25%, while glycerol did not affect the enzyme activity (Table 4).

**Table 4:** Effect of various additives on activity *Aspergillus* sp. DHE7 β-glucosidase

Metal ion/chemical reagent ^a^	Residual activity/% ^b^
Control	100 ± 0.0
Fe^2+^	107 ± 1.2
Zn^2+^	145 ± 5.2
Pb^2+^	85 ± 3.5
Mn^+^	89 ± 3.3
Hg^2+^	71 ± 1.2
Co^+^	65 ± 1.9
Cu^2+^	70 ± 1.3
K^+^	137 ± 2.9
Ca^2+^	108 ± 2.1
Mg^2+^	129 ± 3.5
Na^+^	111 ± 2.7
EDTA	101 ± 1.5
DMSO	125 ± 4.2
Glycerol	102 ± 3.3
β-ME	149 ± 4.6

^a^The concentration of all metal ion/chemical reagent tested in this study was adjusted to 5 mM

^b^The residual activity was evaluated via pre-incubation of the purified β-glucosidase with each chemical at 25 °C for 1 h, followed by measurements of residual activity under standard assay with *p-*NPG. Data are expressed as mean value ± S.D. of triplicate measurements

#### Substrate specificity

The purified extracellular *Aspergillus* sp. DHE7 β-glucosidase hydrolysed a broad range of β-linked substrates; namely *p*-NPG, cellobiose, salicin, lactose, and *p*-NP-β-D-galactopyranoside, but was totally inactive towards the other substrates tested (*p*-nitrophenyl-α-D-glucopyranoside, *p*-nitrophenyl-α-D-galactopyranoside, maltose, sucrose, CMC, and starch) (Table 5).

**Table 5:** Relative rate of hydrolysis of various substrates by *Aspergillus* sp. DHE7 β-glucosidase

Substrate	Relative activity/%^a^
Aryl glycosides	
p-Nitrophenyl-β-D-glucopyranoside	100^b^
p-Nitrophenyl-α-D-glucopyranoside	0.0
p-Nitrophenyl-β-D-galactopyranoside	13
p-Nitrophenyl-α-D-galactopyranoside	0.0
Oligosaccharides	
Cellobiose	100^c^
Lactose	18
Maltose	0.0
Salicin	72
Sucrose	0.0
Polysaccharides	
CMC	0.0
Starch	0.0

^a^The relative hydrolytic rate was determined as percentage of that obtained with *p*-NPG for *p*-NP substrates and percentage of that obtained with cellobiose for di- and polysaccharides

^b^Relative hydrolytic activity of enzyme on *p*-NP substrates was calculated by released *p*-NP concentration

^c^Relative hydrolytic activity of enzyme on oligo- and polysaccharides was calculated by analyzing the released glucose concentration using DNS method

#### Enzyme kinetics

**Fig. 6:**
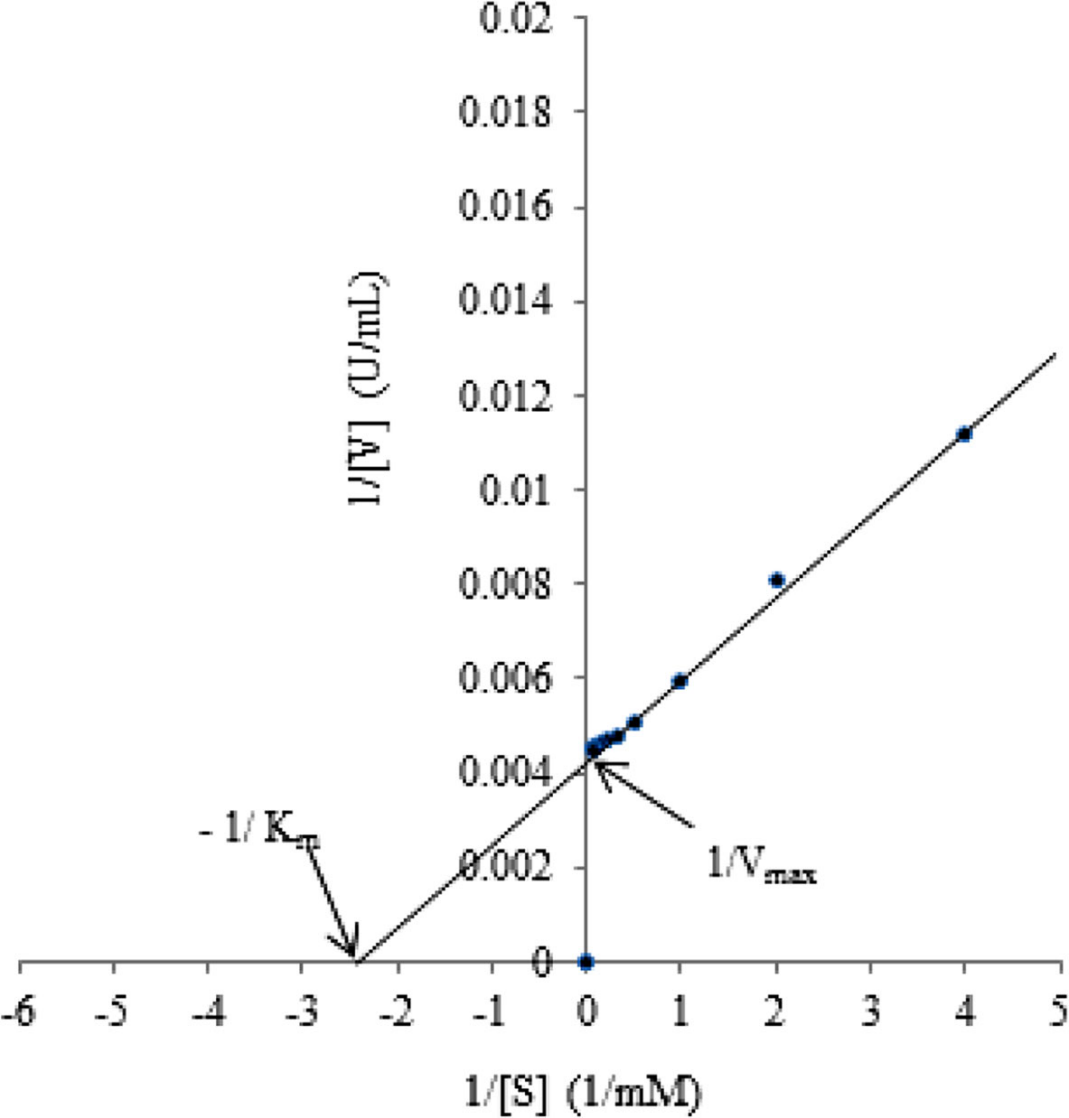
Lineweaver-Burk plot stating hydrolysis rate of different concentrations of *p*-NPG by *Aspergillus* sp. DHE7 β-glucosidase. Enzyme activity was calculated at 60 °C in 50 mM Na-citrate buffer pH 6.0. The values of *K*_*m*_ and *V*_max_ of β-glucosidase were 0.4 mM and 232.6, respectively

Lineweaver-Burk plot was employed to determine the kinetic parameters of *Aspergillus* sp. DHE7 β-glucosidase activity on *p-*NPG under optimal assay conditions (pH 6, 60 °C). Based on the reciprocal double plot given in Fig. 6, measurement of *K*_*m*_ and *V*_max_ were 0.4 mM and 232.6 U/mL, respectively. The lower *K*_*m*_ and higher *V*_max_ of *Aspergillus* sp. DHE7 β-glucosidase enzyme revealed the great affinity of the enzyme against *p*-NPG and the enzyme’s higher catalytic capability.

### Influence of ethanol on *Aspergillus* sp. DHE7 β-glucosidase activity

**Fig. 7:**
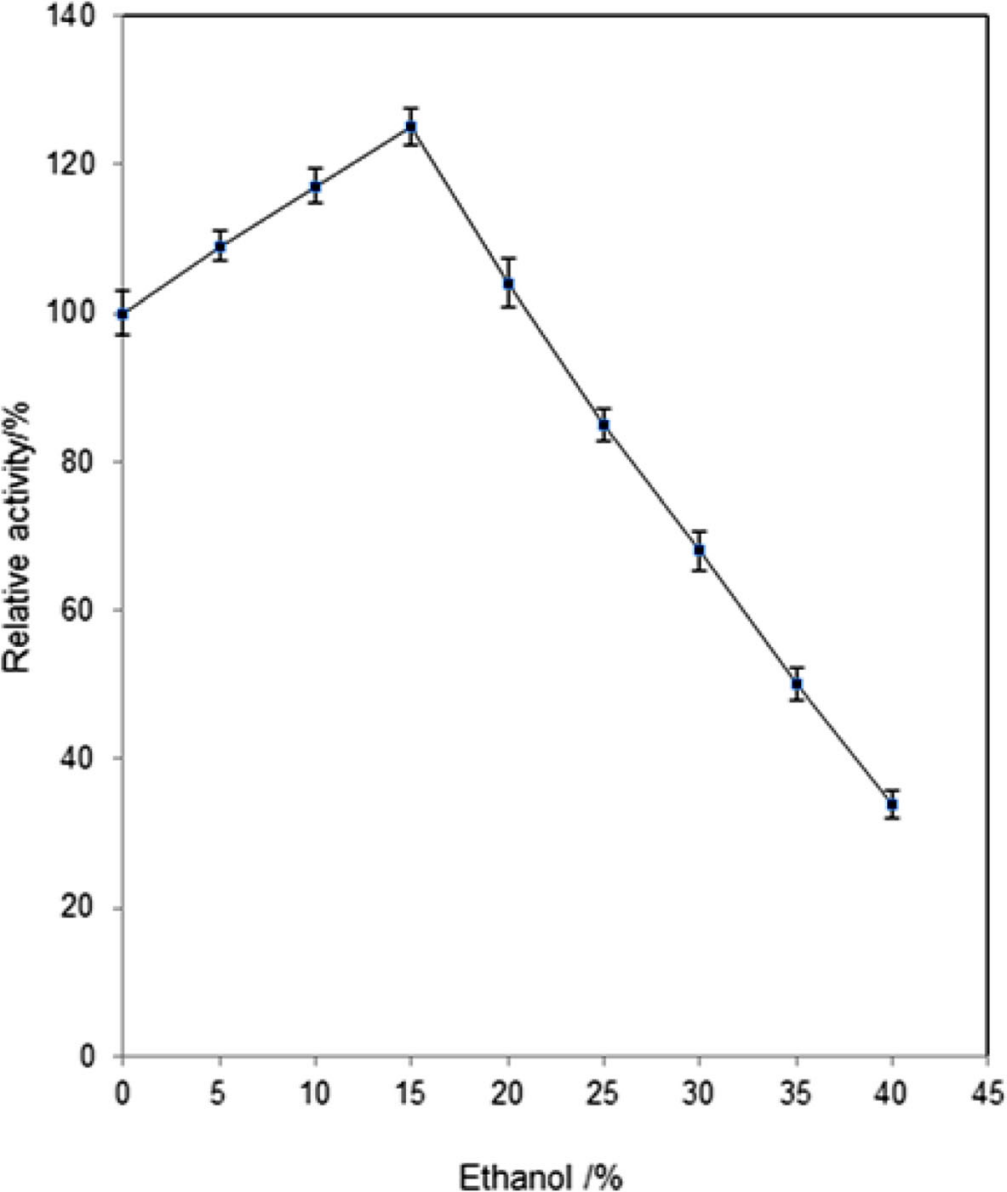
Influence of ethanol on *Aspergillus* sp. DHE 7 β-glucosidase activity. Different concentrations of ethanol (0–40%) were added to the reaction mixture each containing 20 μg of β-glucosidase and 5 mM *p*-NPG in 0.05 M Na-citrate buffer, pH 6.0. All the reaction mixtures were incubated at 60 °C and pH 6.0. The non-treated enzyme activity was regarded as control (100%)

In the current study, β-glucosidase activity on *p*-NPG was evaluated in the presence of ethanol at various concentrations (0–40%, v/v). As evident in Fig. 7, ethanol concentration of 15% improved enzyme activity by 25% compared to the original activity. Moreover, β-glucosidase retained an activity level similar to the control at ethanol concentration of 20%. While raising the concentration of ethanol up to 25% decreased enzymatic activity by 15%.

## Discussion

The enzymatic degradation of cellulose by cellulases has been the focus of several studies for their use in the bioconversions of agricultural wastes, bio-polishing of textiles, the processing of fruit juices, and bioethanol production. Such enzymatic degradation requires the synergistic action between exo-(1, 4)-β-D-glucancellobiohydrolase, endo-(1, 4)-β-D-glucanohydrolase, and β-D-glucosidase [18]. Although numerous microorganisms are known to be able to produce high levels of extracellular cellulases, the best cellulase producers of *Trichoderma* sp. are fairly deficient in β-glucosidase, causing the accumulation of cellobiose, which produces repression and end product inhibition of the enzymes, both of which limit enzyme synthesis and activity [19]. In the current research, 60 fungal isolates were grown under SSF using wheat bran as a substrate. The highest level of extracellular β-glucosidase activity was observed in the culture filtrate of the fungal isolate DHE7 (81.3 ± 4.2 U/g ds), which was much higher than β-glucosidases produced by *Thermoascus aurantiacus* (7.0 U/g ds) and *Lichtheimia ramosa* **(**0.061 U/g ds) [20, 21] grown under SSF. Based on these results, the isolate DHE7 was genetically identified as a new species of *Aspergillus* and the nucleotide information is available in GenBank under the accession number of KX950801.

Most microorganisms possess intracellular and cell-wall bound β-glucosidases [5]. One of the earliest reports in this regard is that of Eberhart and Beck [22] who studied the intracellular localization of β-glucosidase in *Neurospora crassa*. The filamentous fungus *P. decumbens* was also found to produce two intracellular β-glucosidases [23]. While Venturi et al. [24] and Morais et al. [25] reported the production of extracellular β-glucosidase activity from *Chaetomium thermophilum* and *Pleurotus ostreatus* when grown on agro-industrial wastes as substrate. It was reported that the most promising fungi with respect to β-glucosidase production are *Aspergillus* sp. [26]. In addition, the cellulolytic system of *Trichoderma reesei* could be successfully supplemented with β-glucosidase from *Aspergillus* cultures [27].

The utilization of agro-industrial residues as substrate in SSF has gained great interest due to the low-cost production and the reduction in environmental pollution resulted from the accumulation of these wastes. In this study, *Aspergillus* sp. DHE7 could grow on various agro-industrial residues as substrate under SSF; however, jojoba meal was estimated to be the most promising fermentable substrate for β-glucosidase production (116.4 + 2.4 U/g ds). This might be due to the nutritional composition of jojoba meal, which is suitable for the growth of various microorganisms as it contains appropriate amounts of carbohydrates, proteins, fats, fiber, and ashes, favoring enzymes production. In this regard, Xin and Geng [28] reported maximum production of β-glucosidase (61.6 U/g ds) from *T. reesei* when the fungus grown on woodchips for 192 h at 26 °C. Ng et al. [9] observed the highest level of β-glucosidase activity from *P. citrinum* YS40-5 when grown on rice bran for 96 h. While the maximum production of β-glucosidase of 159.3 U/g ds was achieved by *Colletotrichum graminicola* grown in wheat bran containing medium after 168h of incubation [29].

The initial medium pH has a great effect on the growth and the production of β-glucosidase by many microorganisms, as it may affect the permeability of cells and other physiological activities. Most filamentous fungi are known for their capability in growing under a broad range of pH using SSF technique, due to the buffering capacity of these solid substrates [30]. The variation of pH values during the fermentation process depends on the microbial by-products released or the consumed nutrients throughout the cultivation process. Generally, pH values greater than 7 were found to have a negative impact on the fungal growth, thus reducing enzyme production [31]. In the present work, optimum initial medium pH for β-glucosidase production by *Aspergillus* sp. DHE7 was observed in the range of 5.0–7.0 peaking at pH 6.0. This result is closely related to that reported for *A. oryzae* NRRL 3484, which attained its highest level of β-glucosidase yield at pH 5.5 [32]. This slightly acidic pH value was also reported by many investigators; whereas, Grajek [33] and Makropoulou et al. [34] reported that pH 6.0 was the optimal initial pH for β-glucosidases production by *Sporotrichum thermophile* and *Fusarium oxysporum*, respectively. Rajoka et al. [35] also found that the highest yield of this enzyme from *Kluyveromyces marxianus* was occurred at pH 5.5. Results of the current work demonstrated the sensitivity of the enzyme to lower pH values, hence, at initial medium pH of 4.0 only 44.3 ± 2.4 U/g ds of β-glucosidase activity was detected. This finding is similar to that reported for β-glucosidase production from *A. niger* [36]. On the other hand, the reduction in β-glucosidase formation versus alkaline pH was much smaller; whereas, at pH of 8.0, β-glucosidase yield of 86.5 ± 2.1 U/g ds was observed in agreement with the results reported by Hoffman and Wood [37] and Kantham et al. [38].

In SSF, the initial moisture content (IMC) greatly affects the production and secretion of enzymes, depending on the biomass, the microorganism, and the final product. Generally, IMC between 60 and 78% are often used for the production of β-glucosidase by different filamentous fungi [32]. In the current work, maximum β-glucosidase yield of 141.6 ± 4.6 U/g ds was reported at a moisture level of 70%. Similarly, Brijwani et al. [39] found that the moisture level of 70% was the best for the highest β-glucosidase production (10.7 U/g ds) during co-cultivation of *A. oryzae* and *T. reesei* when grown on wheat bran and soybean peel as substrate. In addition, the optimal β-glucosidase production of 70 U/g ds was reported from *Thermoascus aurantiacus* grown in wheat bran medium at a moisture content of 60% [20]. Vu et al. [40] suggested that the presence of free water between the particles of a substrate reduces the substrate porosity; leading to stickiness development and may interfere with gas and heat transfer, while low moisture level tends to reduce nutrient solubility, resulting in an improper swelling that decreases microbial metabolic activity.

Maximum production of β-glucosidase (152.8 ± 4.3 U/g ds) by *Aspergillus* sp. DHE7 was achieved after 72 h of incubation using jojoba meal as a substrate at an initial medium pH of 6.0 and 70% moisture level. However, no significant activity could be detected in the culture filtrates of *Aspergillus* sp. DHE7 after 24 h of incubation. It seemed likely that the cell machinery of the organism during this period is directed towards active vegetative growth and mycelium proliferation. The decreased time of cultivation is considered a key improvement in the fermentation process used by *Aspergillus* sp. DHE7, as the enzyme cost is proportional to the incubation time. In accordance with this result, Mukherjee and Khowala [41] purified an intracellular β-glucosidase from mycelia of *Termitomyces clypeatus* grown in a synthetic medium for 4 days. On the other hand, Juhász et al. [27] reported maximal β-glucosidase production from *A. niger* on the seventh day of incubation. Gonçalves et al. [42] reported optimum β-glucosidase productivity from *L. ramose* after 120 h of incubation. While the highest yield of β-glucosidase of 105.8 U/g ds from *A. fumigatus* was achieved after 96 h of cultivation in wheat bran medium [43].

The optimal temperature for β-glucosidase biosynthesis by *Aspergillus* sp. DHE7 was reported at 35 °C, which is greater than the range of 28–30 °C that is commonly reported for mesophilic microorganisms [39]. This feature promotes the application of this fungal strain in various industrial processes with temperature fluctuations because the control of large-scale fermentation parameters is not as accurate as in the lab. On the other hand, a considerable reduction in β-glucosidase production was observed in cultures grown at 25 °C and 45 °C, which were estimated to be only 55.7% and 46.4% that of the maximum value, respectively. This negative effect might be resulted from the reduction in plasma membrane permeability and metabolic reactions rate at low temperatures, while at high temperatures, the membrane structures collapse and denature structural proteins and enzymes, consequently, resulting in a reduction in enzyme production [44]. Maximum β-glucosidase production of 174.6 ± 5.8 U/g ds from *Aspergillus* sp. DHE7 was reported at an inoculum size of 2.54 × 10^7^ spores/mL. However, a decline in enzyme production was reported with inoculum levels higher or smaller than the optimum level. Whereas at lower inoculum sizes, microbial biomass cannot proliferate rapidly; therefore, the degradation of substrates is slow, subsequently, affecting metabolite production. On the other hand, the inhibitory impact at higher inoculum size might be attributed to the depletion of nutrients and oxygen form the culture medium [45].

*Aspergillus* sp. DHE7 β-glucosidase was purified 29.6-purification fold with 45.2% recovery using 50% ethanol fractionation, followed by DEAE-Sephadex A-50 column then subsequent gel filtration on Sephadex G-100 column. The specific activity of the pure β-glucosidase was 2338.3 U/mg protein, which is much higher than those reported by Chirico and Brown [46] for *Trichoderma reesei* (52 U/mg protein), Bhat et al. [47] for *Sclerotium thermophile* (89 U/mg protein), and Abdel-Naby et al. [48] for *A. niger* A20 (140.35 U/mg protein). The purified enzyme was migrated as only one protein band with an apparent molecular mass of 135 kDa on polyacrylamide gel electrophoresis technique, indicating its homogeneity and purity. This value is favorably comparable to those reported for *A. terreus* ATCC 52430 and its mutant, UNG1-40 (90 kDa and 95 kDa, respectively) [49], *A. niger* KCCM 11239 β-glucosidase (123 kDa) [50] and *Phanerochaete chrysosporium* (114 kDa) [51]. However, the molecular mass of *Aspergillus* sp. DHE7 β-glucosidase is much smaller than those of *Sclerotium thermophile* (240 kDa**)** [46] and *Botrytis cinerea* (350 kDa) [52].

Biochemical characterization of the purified *Aspergillus* sp. DHE7 β-glucosidase indicated that this enzyme displayed its maximum activity at temperature of 60 °C and pH 6.0. A further increase in temperature has resulted in a gradual decrease in enzyme activity, which may result from a conformational change resulting in a loss in the specificity of the active site [53]. It should be noted that temperatures above 50 °C have not been observed routinely for β-glucosidases produced by mesophilic microorganisms. These results are consistent with the findings of Liu et al. [54] on β-glucosidase activity purified form *A. fumigatus* Z5. β-glucosidase purified from the thermophilic fungus *Chaetomium thermophilum* var. *coprophilum* showed its optimal activity at pH 5.5 and 65 °C [24]. Gueguen et al. [52] reported that *Botrytis cinerea* β-glucosidase exhibited optimal catalytic activity at 50 °C and pH 7.0 with citrate-phosphate buffer and 6.5 with phosphate buffer. Optimal temperature of 50–60 °C has also been reported for the enzyme purified from the thermophilic fungus *Humicola grisea* var. *thermoidea* at an optimum pH of 6.0 [55]. While Belancic et al. [52] investigated the optimum activity of *Debaryomyces vanrijiae* β-glucosidase at a lower temperature of 40 °C. Baffi et al. [56] reported that most fungal β-glucosidases have optimum activity at temperatures ranging from 40 to 50 °C, while high catalytic activity in enzymes produced by mesophilic microorganisms is not routinely detected at temperatures above 50 °C. Baffi et al. [56] reported that most fungal β-glucosidases have optimum activity at temperatures ranging from 40 to 50 °C.

The enzyme seems to be also active over a wide range of pH values (4.0–7.0). However, a decline in β-glucosidase activity was observed at pH values below or higher than the optimum pH 6.0. This negative effect may result from the fact that the enzyme is protein in nature and any alteration in the pH value may have a significant effect on the ionic character of its amino or carboxylic groups, which, in turn, may affect the conformation of the enzyme. In addition, the pH reaction may affect the affinity between the enzyme and its substrate [57]. The optimal activity of *Pyrococcus furiosus* β-glucosidase was also exhibited at pH 6.0 but was inactivated at extreme pH values (pH 4.0 and 9.0) [57]. *Fusarium oxysporum* β-glucosidase activity was optimal at pH 5.0 and stable at pH between 4.0 and 7.0 [58]. It is also worth mentioning that the retention of maximum activity over a wide pH range of 3.5–7.0 indicates that β-glucosidase from *Aspergillus* sp. DHE7 may be suitable for the processing of various dairy products.

Measurement of *Aspergillus* sp. DHE7 β-glucosidase thermostability as a function of time and temperature showed that the enzyme was fully stable at temperatures ranging from 30 to 60 °C up to 60 min. A slight decrease in activity was observed at 70 °C after 30 min of incubation. The enzyme retained about 55% of the initial activity at 80 °C after 60 min of incubation. While about 50% of the activity was observed at 90 °C after 15 min of incubation, then activity decreased rapidly, reaching only 23% at the same temperature after 30 min of incubation. These results showed that *Aspergillus* sp. DHE7 β-glucosidase was much more stable than β-glucosidase purified from *T. harzianum*, which remained stable for only 15 min at temperatures below 55 °C and maintained 36% of its initial activity at 60 °C after 15 min [59]. *Sporidiobolus pararoseus* β-glucosidase was able to maintain its activity at 40 °C for 60 min while retaining only 30% of the initial activity at higher temperatures [60].

Glycosidases may be divided into three groups on the basis of substrate specificity: (i) aryl-β-glucosidase (which hydrolyzes exclusively aryl-β-glucosides), (ii) cellobiase (which hydrolyzes only cellobiose and short-chain cello-dextrins), and (iii) broad specificity of β-glucosidases (which show activity on both substrate types). However, the relative activity against cellobiose and aryl-β-D-glucosides depends on the source of the enzyme [61]. Plant et al. [62] reported that β-glucosidase preferred aryl glycoside substrates due to high electrophilicity of aglycone moiety, which improves the stability of the *ortho* or *para*-nitrophenoxide anion produced during the initial stage of catalysis. In current research, β-glucosidase purified from *Aspergillus* sp. DHE7 showed a broad specificity type as it exhibited high reactivity towards *p*-nitrophenyl-β-D-glucopyranoside, cellobiose, salicin, lactose and *p*-NP-β-D-galactopyranoside. These results suggested that *Aspergillus* sp. DHE7 β-glucosidase is a broad spectrum enzyme and may have a potential application in various biotechnological fields. β-glucosidase purified from *Talaromyces thermophiles* showed affinity against lactose, maltose, and cellobiose with 75, 61, and 6% relative activity, respectively [4]. The broad substrate specificity of β-glucosidase can be attributed to the wide and extended cavity structure surrounding the active-center cavity as determined by homology modelling [63]. Generally, the high specificity of *Aspergillus* sp. DHE7 β-glucosidase justifies its suitability for the enrichment of cellulolytic complexes defective in β-glucosidase, especially that of *Trichoderma*.

The effect of substrate concentration on the velocity of the reaction was tested with *p*-NPG to determine whether or not β-glucosidase is an allosteric enzyme and also to calculate the apparent *K*_*m*_ value. Obtaining hyperbolic rather than sigmoid saturation kinetics indicates that *Aspergillus* sp. DHE7 β-glucosidase does not appear to be an allosteric enzyme. The apparent *K*_*m*_ and *V*_max_ values were 0.4 mM and 232.6 U/mL, respectively. The lower *K*_*m*_ and higher *V*_max_ of *Aspergillus* sp. DHE7 β-glucosidase enzyme revealed the high affinity of the enzyme toward *p*-NPG and the higher catalytic capacity of the enzyme. β-glucosidase produced by thermophilic fungus *Chaetomium thermophilum* hydrolysed cellobiose and *p*-NPG with apparent *K*_*m*_ values of 3.13 mM and 0.76 mM, respectively [24]. In addition, Saha and Bothast [64] reported the production of β-glucosidase by *Candida peltata* which hydrolysed *p*-NPG and cellobiose with *K*_*m*_ values of 2.3 and 66 mM, respectively. *K*_*m*_ values between 0.1 and 44 mM *p*-NPG have been reported for fungal β-glucosidases, and these variations could be related to differences in enzyme assay conditions and substrate preferences [65].

Among the different metal ions and chemical reagents tested, an increase in enzyme activity was reported with Zn^2+^, K^+^, Mg^2+^, and SH-modifying β-mercaptoethanol reagent, while a slight increase in enzyme activity was observed with Na^+^, Ca^2+^, and Fe^2+^. On the other hand, moderate inhibition was observed by Mn^+^, Pb^2+^, Hg^2+^, Cu^2^, and Co^+^. The thiol group inhibitor Ag^+^ had strongly inhibited B-glucosidase activity, indicating that SH-groups are essential for enzymatic activity and may be located at the active site [66]. The metal chelating agent EDTA did not inhibit β-glucosidase activity, suggesting that the enzyme is not a metalloprotein. In accordance with these results**,** Shipkowski and Brenchley [67] found that EDTA treatment had no effect on β-glucosidase produced by *Paenibacillus* sp. Strain C7. The activity of β-glucosidases purified from different fungi have been was found to be strongly activated by Zn^2+^ [68] while inhibited by Hg^2+^, Cu^2+^, Ag^+^, Hg^2+^, Pb^2+^, and Cd^2+^ [69]. Zhang et al. [70] investigated that Ca^2+^, Mg^2+^, and Zn^2+^ had no significant impact on the activity of *A. oryzae* β-glucosidase while Cu^2+^ ion strongly inhibited the enzyme activity. In addition, the inhibition of *A. ornatus* β-glucosidase in the occurrence of Ag^2+^ and Fe^2+^ was reported by Yeoh et al. [66]. Peralta et al. [55] reported that the addition of different metal ions (Mg^2+^, Ca^2+^, Co^2+^, Al^2+^, Cu^2+^, Zn^2+^, and Mn^2+^) had no effect on β-glucosidase activity**,** while Hg^2+^ and Ag^2+^ had virtually inhibited the activity.

In studies with β-glucosidase, it is important to determine the impact of ethanol on β-glucosidase activity as this enzyme is exposed to significant concentrations of alcohol in a number of industrial applications. In addition, β-glucosidase can catalyze under certain conditions the transglycosylation reaction in the presence of ethanol [71]. In the current study, the β-glucosidase activity on *p*-NPG was evaluated in the presence of ethanol up to 40% (v/v). Data revealed that concentrations of ethanol up to 15% improved the activity of the enzyme by 25% compared to the original activity. Moreover, β-glucosidase could retain an activity level similar to control at a concentration of 20% ethanol. Taking into consideration that the final concentration of ethanol produced by conventional methods in fermented broths is about 10%, it is suggested that the current *Aspergillus* sp. DHE7 *β*-glucosidase is fairly stable for use in ethanol-containing industrial processes and in saccharification processes for bioethanol production [72]. Arévalo et al. [73] investigated that an improvement in the enzyme catalytic potential in the presence of ethanol might be attributed to the activity of glucosyl transferase. During the enzymatic catalysis, ethanol can enhance the reaction rate by acting as an acceptor of glycosyl residues. Transglycosylation and hydrolysis proceed along the same biochemical pathway, varying only in the final acceptor nature [74]. In addition, Mateo and Di Stefano [75] reported that changes in medium polarity induced by alcohols could stabilize the conformation of the enzyme.

## Conclusions

In conclusion, the current β-glucosidase purified from the newly isolated terrestrial fungus *Aspergillus* sp. DHE7 shows great potential as an industrial source of β-glucosidase. The enzyme is extremely stable and is not significantly affected by cations and could be produced efficiently on a low-cost medium, i.e., agro-industrial waste. One of its most important attributes is that its pH and temperature optimum are consistent with those reported for *Trichoderma* cellulase, indicating the potential for supplementation of *Trichoderma* cellulase with *Aspergillus* sp. DHE7 β-glucosidase. In addition, the activity of the current β-glucosidase was potentiated up to 25% by 15% ethanol while retaining its initial activity in solutions containing 20% ethanol, suggesting the use of *Aspergillus* sp. DHE7 β-glucosidase in lignocellulosic biomass hydrolysis for biofuel production and various biotechnological applications. It is worth mentioning that quantitative real-time polymerase chain reaction (qRT-PCR) for the amplified ITS region of *Aspergillus* sp. DHE7 will be studied in the future research to confirm the results of enzyme activity through quantification of the related β-glucosidase genes.

## Supplementary Information

**Additional file 1: S. 1** Results of PCR amplification. **S. 2** Aligned sequence of the fungal isolate DHE7 18S rRNA gene. **S. 3** The most closely related *Aspergillus* species and their percentages of identity. **S. 4** Multiple-sequence alignment of the sequences of amplified targeted ITS region.

## Abbreviations

*p*-NPG: *p*-Nitrophenyl-β-D-glucopyranoside
*p*-NP: *p*-Nitrophenol
BSA: Bovine serum albumin
PDA: Potato dextrose agar
PDB: Potato dextrose broth
DNS: 3,5-dinitrosalicylic acid
SSF: Solid-state fermentation
β-GL: β-glucosidase
SDS-PAGE: Sodium dodecyl sulfate polyacrylamide gel electrophoresis
DMSO: Dimethyl sulfoxide
EDTA: Ethylenediaminetetraacetic acid
UV: Ultra violet
qRT-PCR: Quantitative real-time polymerase chain reaction
