# Supplementary material for: Optimization of extracellular ethanol-tolerant β-glucosidase production from a newly isolated Aspergillus sp. DHE7 via solid state fermentation using jojoba meal as substrate: purification and biochemical characterization for biofuel preparation
Source: J Genet Eng Biotechnol. 2021 Mar 24;19:45. doi: 10.1186/s43141-021-00144-z (PMC7991022; doi:10.1186/s43141-021-00144-z)
Supplement: Supplementary file 1 — Additional file 1: S. 1 Results of PCR amplification. S. 2 Aligned sequence of the fungal isolate DHE7 18S rRNA gene. S. 3 The most closely related Aspergillus species and their percentages of identity. S. 4 Multiple-sequence alignment of the sequences of amplified targeted ITS region. [file 43141_2021_144_MOESM1_ESM.pdf]

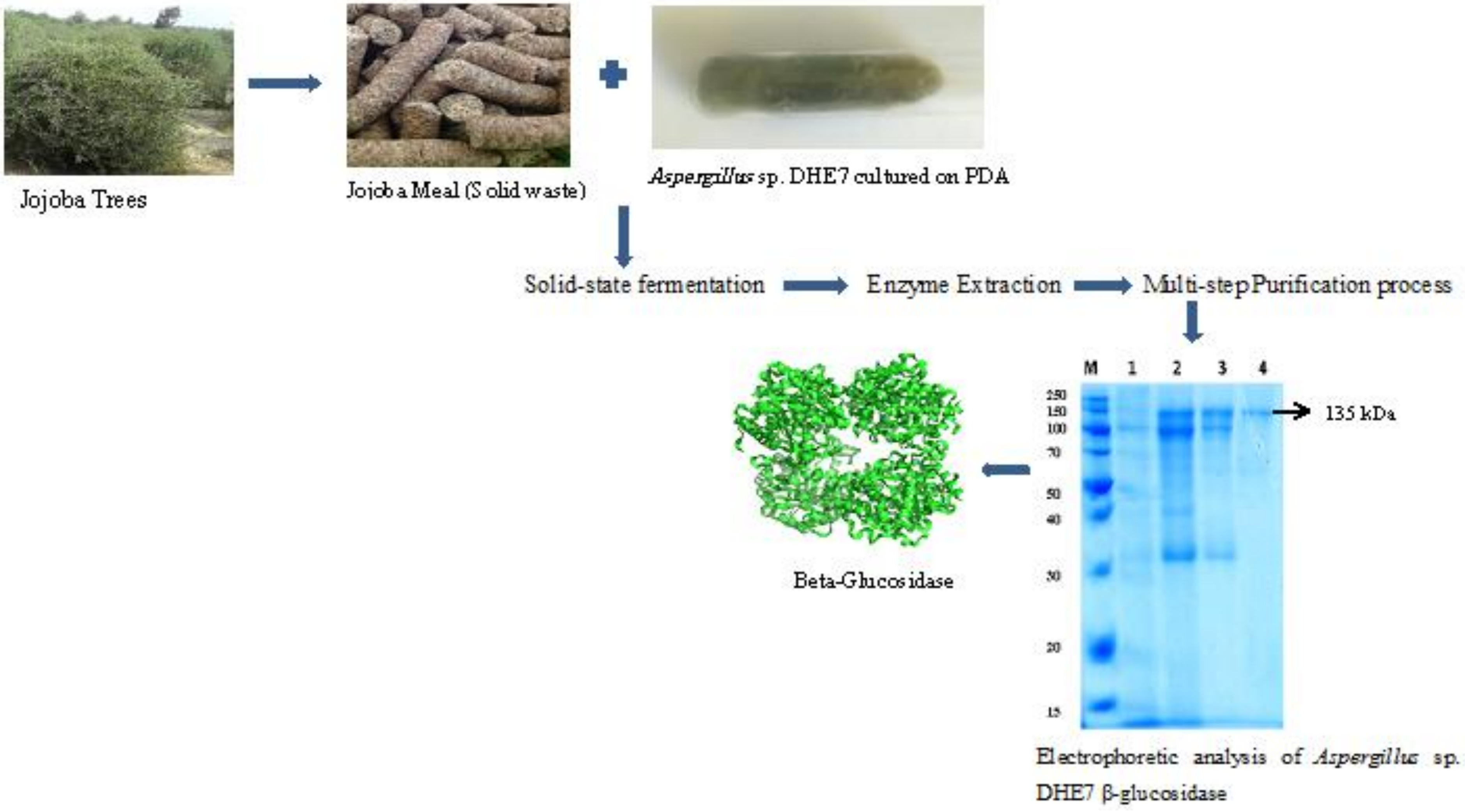

Graphical abstract

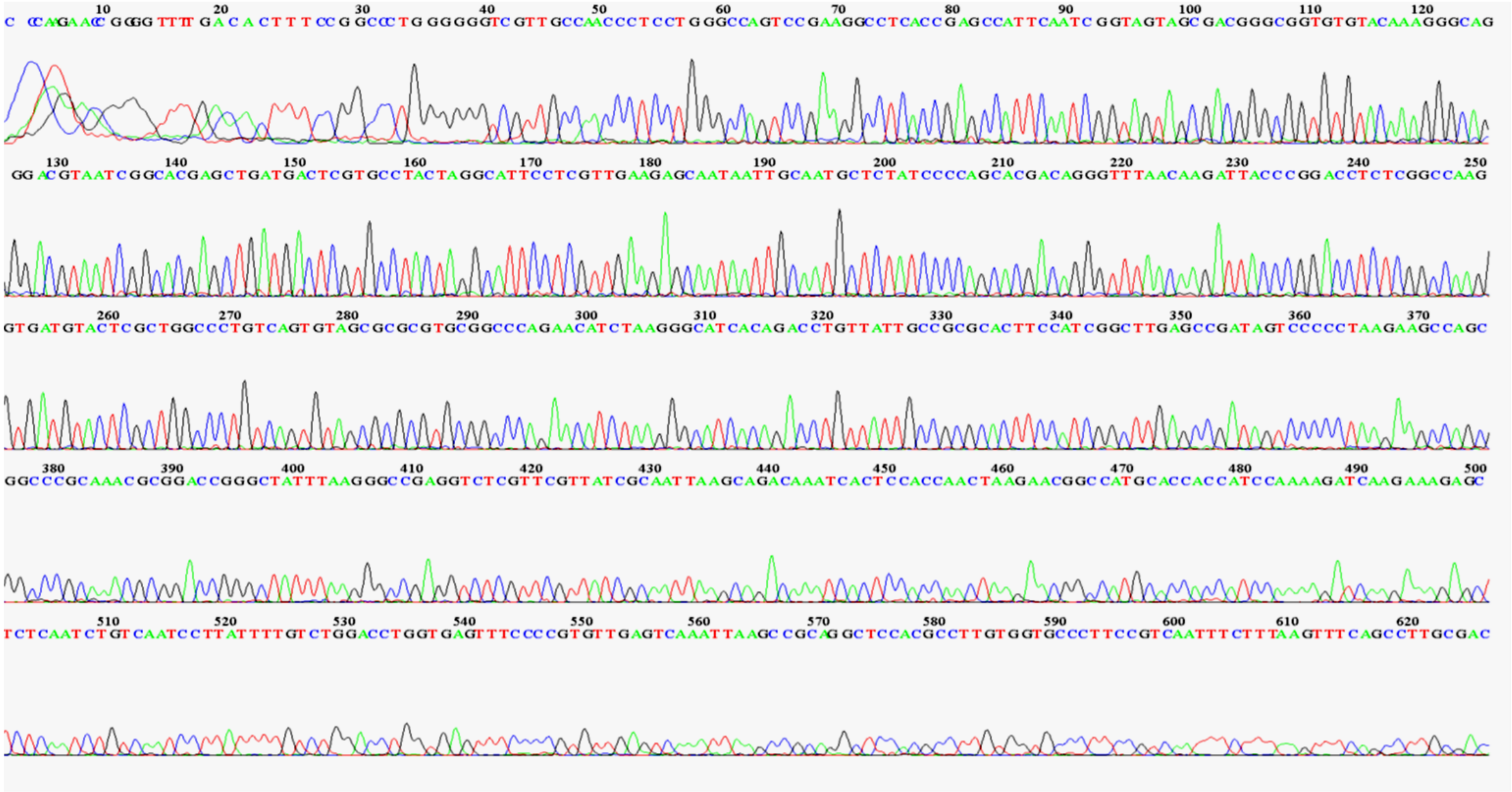

S. 1 Results of PCR amplification

AGCTCTCTATTCTAGGATGCTCTGACATACGGGAAGAGTACCATTTAATCTAATCGATCACTGGAGGCC
AGTGTCTGGTGCCAGCAGCCGCGGTAATTCCAGTTCCAATTAGCGATATATAAAGTTGTTGCAGTAAAA
AGCTCGTAGTTGACCTTTGGGTCATGACTGGCCGTCCCGCCTCACCAGCGAGGTACTGGTCCGGACTGA
CCTTTCCTTTTAGGGGACCTCATGGCCTTTCACTGGACTGTGGAGGGACAAGATCTTTTCCTGTGGAAA
AAAATTAGAGTGTTCAAAGCAAGGCCTTTGCTCGAATACATTAGCATTGAAATATAGAATAGGACGTGC
GGTTTCTATTTTGTTGAGTTTCTAGGACCGCCGTAATGATTAATAGGGATAGTCGGGGGCGTCAGTATT
CAGCTGTCAGAGGTGGAAATTCTAGGATTTGCTGAAAGACTAACTACTGCGAAAGCATTCGCCAAGGAT
GTTTTCATTAATCAGGGAACGAAAGTTAGGGGATCGAAGACGATCAGATACCGTCGTAGTCTTAACCAT
AAACTATGCCGACTAGGGATCGGGCGGTGTTTCTATGATGACCCGCTCGGCACCTTACGAGAAATCAAA
GTTTTTGGGTTCTGGGGGGAGTATGGTCGCAAGGCTGAAACTTAAAGAAATTGACGGAAGGGCACCACA
AGGCGTGGAGCCTGCGGCTTAATTTGACTCAACACGGGGAAACTCACCAGGTCCAGACAAAATAAGGAT
TGACAGATTGAGAGCTCTTTCTTGATCTTTTGGATGGTGGTGCATGGCCGTTCTTAGTTGGTGGAGTGA
TTTGTCTGCTTAATTGCGATAACGAACGAGACCTCGGCCCTTAAATAGCCCGGTCCGCGTTTGCGGGCC
GCTGGCTTCTTAGGGGGACTATCGGCTCAAGCCGATGGAAGTGCGCGGCAATAACAGGTCTGTGATGCC
CTTAGATGTTCTGGGCCGCACGCGCGCTACACTGACAGGGCCAGCGAGTACATCACCTTGGCCGAGAGG
TCCGGGTAATCTTGTTAAACCCTGTCGTGCTGGGGATAGAGCATTGCAATTATTGCTCTTCAACGAGGA
ATGCCTAGTAGGCACGAGTCATCAGCTCGTGCCGATTACGTCCCTGCCCTTTGTACACACCGCCCGTCG
CTACTACCGATTGAATGGCTCGGTGAGGCCTTCGGACTGGCCCAGGAGGGTTGGCAACGACCCCCCAGG
GCCGGAAAGTGTCAAAACCCCGGTTCTTGGG

S. 2 Aligned sequence of the fungal isolate DHE7 18S rRNA gene

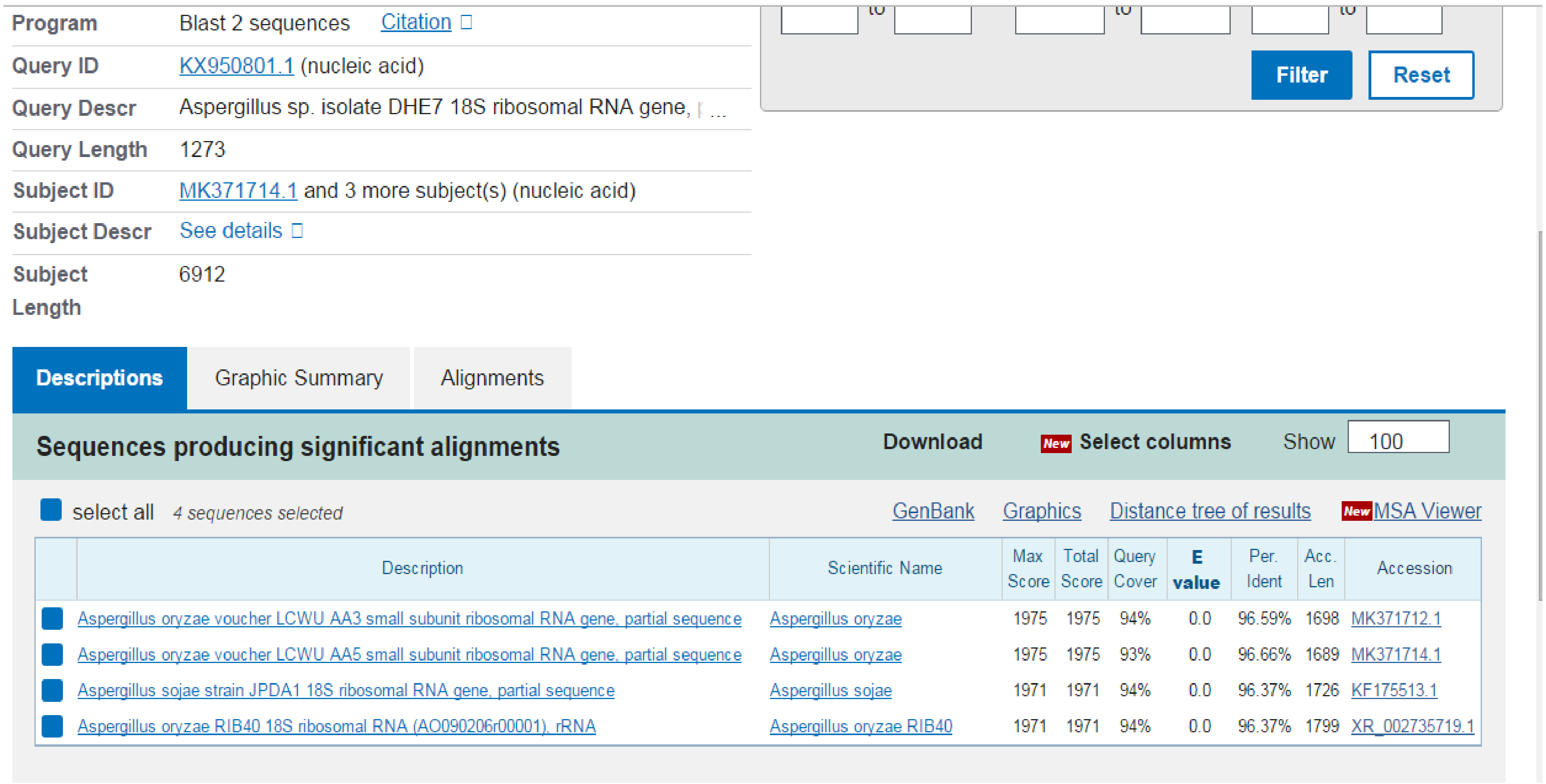

S. 3 The most closely related *Aspergillus* species and their percentages of identity

```
KX950801.1          ---AGCTCTCTATTCTAGGATGCTCTGACATACGGGAAGAGTACCATTTAATCT---AAT      54
MK371712.1          TACGGGGCTCT--TTTG----GGTCTCGTAATTGGAATGAGTACAATCTAAATCCCTTAA      485
MK371714.1          TACGGGGCTCT--TTTG----GGTCTCGTAATTGGAATGAGTACAATYTAAATCCCTTAA      485
XR_002735719.1      TACGGGGCTCT--TTTG----GGTCTCGTAATTGGAATGAGTACAATCTAAATCCCTTAA      533
KF175513.1          TACGGGGCTCT--TTTG----GGTCTCGTAATTGGAATGAGTACAATCTAAATCCCTTAA      509
                        *  ****  * *     * ***   *   ** * ****** ** ***       *

KX950801.1          CG----ATCACTGGAGGCCAGTGTCTGGTGCCAGCAGCCGCGGTAATTCCAGTTCCAATT      110
MK371712.1          CGAGGAACAATTGGAGGGCA-AGTCTGGTGCCAGCAGCCGCGGTAATTCCAGCTCCAATA      544
MK371714.1          CGAGGAACAATTGGAGGGCA-AGTCTGGTGCCAGCAGCCGCGGTAATTCCAGCTCCAATA      544
XR_002735719.1      CGAGGAACAATTGGAGGGCA-AGTCTGGTGCCAGCAGCCGCGGTAATTCCAGCTCCAATA      592
KF175513.1          CGAGGAACAATTGGAGGGCA-AGTCTGGTGCCAGCAGCCGCGGTAATTCCAGCTCCAATA      568
                    **    *  * ****** **  ****************************** ******

KX950801.1          AGCGATATATAAAGTTGTTGCAGT-AAAAAGCTCGTAGTTGACCTTTGGGTCATGACTGG      169
MK371712.1          GCGTATA-TTAAAGTTGTTGCAGTTAAAAAGCTCGTAGTTGAACCTTGGGTC-TGGCTGG      602
MK371714.1          GCGTATA-TTAAAGTTGTTGCAGTTAAAAAGCTCGTAGTTGAACCTTGGGTC-TGGCTGG      602
XR_002735719.1      GCGTATA-TTAAAGTTGTTGCAGTTAAAAAGCTCGTAGTTGAACCTTGGGTC-TGGCTGG      650
KF175513.1          GCGTATA-TTAAAGTTGTTGCAGTTAAAAAGCTCGTAGTTGAACCTTGGGTC-TGGCTGG      626
                        ***  *************** ***************** * ******* ** ****

KX950801.1          CCGTCCCGCCTCACCAGCGAGGTACTGGTCCGGACTGACCTTTCCTTTTAGGGGACCTCA      229
MK371712.1          CCGGTCCGCCTCACCG-CG-AGTACTGGTCCGGCTGGACCTTTCCTTCTGGGGAACCTCA      660
MK371714.1          CCGGTCCGCCTCACCG-CG-AGTACTGGTCCGGCTGGACCTTTCCTTCTGGGGAACCTCA      660
XR_002735719.1      CCGGTCCGCCTCACCG-CG-AGTACTGGTCCGGCTGGACCTTTCCTTCTGGGGAACCTCA      708
KF175513.1          CCGGTCCGCCTCACCG-CG-AGTACTGGTCCGGCTGGACCTTTCCTTCTGGGGAACCTCA      684
                    ***  **********  **  ************   *********** * *** ******

KX950801.1          TGGCCTTTCACTGGACTGTGGAGGGACAAGATCTTTTCCTGTGGAAAAAAATTAGAGTGT      289
MK371712.1          TGGCCT-TCACTGGCTGTGGGGGGAACCAGGACTTTTACTGTGA--AAAAATTAGAGTGT      717
MK371714.1          TGGCCT-TCACTGGCTGTGGGGGGAACCAGGACTTTTACTGTGA--AAAAATTAGAGTGT      717
XR_002735719.1      TGGCCT-TCACTGGCTGTGGGGGGAACCAGGACTTTTACTGTGA--AAAAATTAGAGTGT      765
KF175513.1          TGGCCT-TCACTGGCTGTGGGGGGAACCAGGACTTTTACTGTGA--AAAAATTAGAGTGT      741
                    ****** *******     ** ** ** **  ***** *****   **************

KX950801.1          TCAAAGCAAGGCCTTTGCTCGAATACATTAGCATTGAAATATAGAATAGGACGTGCGGTT      349
MK371712.1          TCAAAGCA-GGCCTTTGCTCGAATACATTAGCATGGAATAATAGAATAGGACGTGCGGT-      775
MK371714.1          TCAAAGCA-GGCCTTTGCTCGAATACATTAGCATGGAATAATAGAATAGGACGTGCGGT-      775
XR_002735719.1      TCAAAGCA-GGCCTTTGCTCGAATACATTAGCATGGAATAATAGAATAGGACGTGCGGT-      823
KF175513.1          TCAAAGCA-GGCCTTTGCTCGAATACATTAGCATGGAATAATAGAATAGGACGTGCGGT-      799
                    ******** ************************* ***  *******************

KX950801.1          TCTATTTTGTTGAGTTTCTAGGACCGCCGTAATGATTAATAGGGATAGTCGGGGGCGTCA      409
MK371712.1          TCTATTTTGTTG-GTTTCTAGGACCGCCGTAATGATTAATAGGGATAGTCGGGGGCGTCA      834
MK371714.1          TCTATTTTGTTG-GTTTCTAGGACCGCCGTAATGATTAATAGGGATAGTCGGGGGCGTCA      834
XR_002735719.1      TCTATTTTGTTG-GTTTCTAGGACCGCCGTAATGATTAATAGGGATAGTCGGGGGCGTCA      882
KF175513.1          TCTATTTTGTTG-GTTTCTAGGACCGCCGTAATGATTAATAGGGATAGTCGGGGGCGTCA      858
                    ************ ***********************************************

KX950801.1          GTATTCAGCTGTCAGAGGTGGAAATTCTAGGATTTGCTGAAAGACTAACTACTGCGAAAG      469
MK371712.1          GTATTCAGCTGTCAGAGGTG-AAATTCTTGGATTTGCTGA-AGACTAACTACTGCGAAAG      892
MK371714.1          GTATTCAGCTGTCAGAGGTG-AAATTCTTGGATTTGCTGA-AGACTAACTACTGCGAAAG      892
XR_002735719.1      GTATTCAGCTGTCAGAGGTG-AAATTCTTGGATTTGCTGA-AGACTAACTACTGCGAAAG      940
KF175513.1          GTATTCAGCTGTCAGAGGTG-AAATTCTTGGATTTGCTGA-AGACTAACTACTGCGAAAG      916
                    ******************** ******* *********** *******************

KX950801.1          CATTCGCCAAGGATGTTTTCATTAATCAGGGAACGAAAGTTAGGGGATCGAAGACGATCA      529
MK371712.1          CATTCGCCAAGGATGTTTTCATTAATCAGGGAACGAAAGTTAGGGGATCGAAGACGATCA      952
MK371714.1          CATTCGCCAAGGATGTTTTCATTAATCAGGGAACGAAAGTTAGGGGATCGAAGACGATCA      952
XR_002735719.1      CATTCGCCAAGGATGTTTTCATTAATCAGGGAACGAAAGTTAGGGGATCGAAGACGATCA      1000
KF175513.1          CATTCGCCAAGGATGTTTTCATTAATCAGGGAACGAAAGTTAGGGGATCGAAGACGATCA      976
                    ************************************************************

KX950801.1          GATACCGTCGTAGTCTTAACCATAAACTATGCCGACTAGGGATCGGGCGGTGTTTCTATG      589
MK371712.1          GATACCGTCGTAGTCTTAACCATAAACTATGCCGACTAGGGATCGGGCGGTGTTTCTATG      1012
MK371714.1          GATACCGTCGTAGTCTTAACCATAAACTATGCCGACTAGGGATCGGGCGGTGTTTCTATG      1012
XR_002735719.1      GATACCGTCGTAGTCTTAACCATAAACTATGCCGACTAGGGATCGGGCGGTGTTTCTATG      1060
KF175513.1          GATACCGTCGTAGTCTTAACCATAAACTATGCCGACTAGGGATCGGGCGGTGTTTCTATG      1036
                    ************************************************************

KX950801.1          ATGACCCGCTCGGCACCTTACGAGAAATCAAAGTTTTTGGGTTCTGGGGGGAGTATGGTC      649
MK371712.1          ATGACCCGCTCGGCACCTTACGAGAAATCAAAGTTTTTGGGTTCTGGGGGGAGTATGGTC      1072
MK371714.1          ATGACCCGCTCGGCACCTTACGAGAAATCAAAGTTTTTGGGTTCTGGGGGGAGTATGGTC      1072
XR_002735719.1      ATGACCCGCTCGGCACCTTACGAGAAATCAAAGTTTTTGGGTTCTGGGGGGAGTATGGTC      1120
KF175513.1          ATGACCCGCTCGGCACCTTACGAGAAATCAAAGTTTTTGGGTTCTGGGGGGAGTATGGTC      1096
                    ************************************************************

KX950801.1          GCAAGGCTGAAACTTAAAGAAATTGACGGAAGGGCACCACAAGGCGTGGAGCCTGCGGCT      709
MK371712.1          GCAAGGCTGAAACTTAAAGAAATTGACGGAAGGGCACCACAAGGCGTGGAGCCTGCGGCT      1132
MK371714.1          GCAAGGCTGAAACTTAAAGAAATTGACGGAAGGGCACCACAAGGCGTGGAGCCTGCGGCT      1132
XR_002735719.1      GCAAGGCTGAAACTTAAAGAAATTGACGGAAGGGCACCACAAGGCGTGGAGCCTGCGGCT      1180
KF175513.1          GCAAGGCTGAAACTTAAAGAAATTGACGGAAGGGCACCACAAGGCGTGGAGCCTGCGGCT      1156
                    ************************************************************

KX950801.1          TAATTTGACTCAACACGGGGAAACTCACCAGGTCCAGACAAAATAAGGATTGACAGATTG      769
MK371712.1          TAATTTGACTCAACACGGGGAAACTCACCAGGTCCAGACAAAATAAGGATTGACAGATTG      1192
MK371714.1          TAATTTGACTCAACACGGGGAAACTCACCAGGTCCAGACAAAATAAGGATTGACAGATTG      1192
XR_002735719.1      TAATTTGACTCAACACGGGGAAACTCACCAGGTCCAGACAAAATAAGGATTGACAGATTG      1240
KF175513.1          TAATTTGACTCAACACGGGGAAACTCACCAGGTCCAGACAAAATAAGGATTGACAGATTG      1216
                    ************************************************************
```

```
KX950801.1        AGAGCTCTTTCTTGATCTTTTGGATGGTGGTGCATGGCCGTTCTTAGTTGGTGGAGTGAT    829
MK371712.1        AGAGCTCTTTCTTGATCTTTTGGATGGTGGTGCATGGCCGTTCTTAGTTGGTGGAGTGAT    1252
MK371714.1        AGAGCTCTTTCTTGATCTTTTGGATGGTGGTGCATGGCCGTTCTTAGTTGGTGGAGTGAT    1252
XR_002735719.1    AGAGCTCTTTCTTGATCTTTTGGATGGTGGTGCATGGCCGTTCTTAGTTGGTGGAGTGAT    1300
KF175513.1        AGAGCTCTTTCTTGATCTTTTGGATGGTGGTGCATGGCCGTTCTTAGTTGGTGGAGTGAT    1276
                  ************************************************************

KX950801.1        TTGTCTGCTTAATTGCGATAACGAACGAGACCTCGGCCCTTAAATAGCCCGGTCCGCGTT    889
MK371712.1        TTGTCTGCTTAATTGCGATAACGAACGAGACCTCGGCCCTTAAATAGCCCGGTCCGCGTT    1312
MK371714.1        TTGTCTGCTTAATTGCGATAACGAACGAGACCTCGGCCCTTAAATAGCCCGGTCCGCGTT    1312
XR_002735719.1    TTGTCTGCTTAATTGCGATAACGAACGAGACCTCGGCCCTTAAATAGCCCGGTCCGCGTT    1360
KF175513.1        TTGTCTGCTTAATTGCGATAACGAACGAGACCTCGGCCCTTAAATAGCCCGGTCCGCGTT    1336
                  ************************************************************

KX950801.1        TGCGGGCCGCTGGCTTCTTAGGGGGACTATCGGCTCAAGCCGATGGAAGTGCGCGGCAAT    949
MK371712.1        TGCGGGCCGCTGGCTTCTTAGGGGGACTATCGGCTCAAGCCGATGGAAGTGCGCGGCAAT    1372
MK371714.1        TGCGGGCCGCTGGCTTCTTAGGGGGACTATCGGCTCAAGCCGATGGAAGTGCGCGGCAAT    1372
XR_002735719.1    TGCGGGCCGCTGGCTTCTTAGGGGGACTATCGGCTCAAGCCGATGGAAGTGCGCGGCAAT    1420
KF175513.1        TGCGGGCCGCTGGCTTCTTAGGGGGACTATCGGCTCAAGCCGATGGAAGTGCGCGGCAAT    1396
                  ************************************************************

KX950801.1        AACAGGTCTGTGATGCCCTTAGATGTTCTGGGCCGCACGCGCGCTACACTGACAGGGCCA    1009
MK371712.1        AACAGGTCTGTGATGCCCTTAGATGTTCTGGGCCGCACGCGCGCTACACTGACAGGGCCA    1432
MK371714.1        AACAGGTCTGTGATGCCCTTAGATGTTCTGGGCCGCACGCGCGCTACACTGACAGGGCCA    1432
XR_002735719.1    AACAGGTCTGTGATGCCCTTAGATGTTCTGGGCCGCACGCGCGCTACACTGACAGGGCCA    1480
KF175513.1        AACAGGTCTGTGATGCCCTTAGATGTTCTGGGCCGCACGCGCGCTACACTGACAGGGCCA    1456
                  ************************************************************

KX950801.1        GCGAGTACATCACCTTGGCCGAGAGGTCCGGGTAATCTTGTTAAACCCTGTCGTGCTGGG    1069
MK371712.1        GCGAGTACATCACCTTGGCCGAGAGGTCCGGGTAATCTTGTTAAACCCTGTCGTGCTGGG    1492
MK371714.1        GCGAGTACATCACCTTGGCCGAGAGGTCCGGGTAATCTTGTTAAACCCTGTCGTGCTGGG    1492
XR_002735719.1    GCGAGTACATCACCTTGGCCGAGAGGTCCGGGTAATCTTGTTAAACCCTGTCGTGCTGGG    1540
KF175513.1        GCGAGTACATCACCTTGGCCGAGAGGTCCGGGTAATCTTGTTAAACCCTGTCGTGCTGGG    1516
                  ************************************************************

KX950801.1        GATAGAGCATTGCAATTATTGCTCTTCAACGAGGAATGCCTAGTAGGCACGAGTCATCAG    1129
MK371712.1        GATAGAGCATTGCAATTATTGCTCTTCAACGAGGAATGCCTAGTAGGCACGAGTCATCAG    1552
MK371714.1        GATAGAGCATTGCAATTATTGCTCTTCAACGAGGAATGCCTAGTAGGCACGAGTCATCAG    1552
XR_002735719.1    GATAGAGCATTGCAATTATTGCTCTTCAACGAGGAATGCCTAGTAGGCACGAGTCATCAG    1600
KF175513.1        GATAGAGCATTGCAATTATTGCTCTTCAACGAGGAATGCCTAGTAGGCACGAGTCATCAG    1576
                  ************************************************************

KX950801.1        CTCGTGCCGATTACGTCCCTGCCCTTTGTACACACCGCCCGTCGCTACTACCGATTGAAT    1189
MK371712.1        CTCGTGCCGATTACGTCCCTGCCCTTTGTACACACCGCCCGTCGCTACTACCGATTGAAT    1612
MK371714.1        CTCGTGCCGATTACGTCCCTGCCCTTTGTACACACCGCCCGTCGCTACTACCGATTGAAT    1612
XR_002735719.1    CTCGTGCCGATTACGTCCCTGCCCTTTGTACACACCGCCCGTCGCTACTACCGATTGAAT    1660
KF175513.1        CTCGTGCCGATTACGTCCCTGCCCTTTGTACACACCGCCCGTCGCTACTACCGATTGAAT    1636
                  ************************************************************

KX950801.1        GGCTCGGTGAGGCCTTCGGACTGGCCCAGGAGGGTTGGCAACGACCCCCCAGGGCCGGAA    1249
MK371712.1        GGCTCGGTGAGGCCTTCGGACTGGCCCAGGAGGGTTGGCAACGACCCCCCAGGGCCGGAA    1672
MK371714.1        GGCTCGGTGAGGCCTTCGGACTGGCCCAGGAGGGTTGGCAACGACCCCCCAGGGCCGGAA    1672
XR_002735719.1    GGCTCGGTGAGGCCTTCGGACTGGCCCAGGAGGGTTGGCAACGACCCCCCAGGGCCGGAA    1720
KF175513.1        GGCTCGGTGAGGCCTTCGGACTGGCCCAGGAGGGTTGGCAACGACCCCCCAGGGCCGGAA    1696
                  ************************************************************

KX950801.1        AGTGTCAAAACCCCGGTTCTTGGG------------------------------------    1273
MK371712.1        AGCTGTCAAAATCACGTCCTCGCTCT----------------------------------    1698
MK371714.1        AGTGT--CAG---CCGTCATGT--------------------------------------    1689
XR_002735719.1    AGTTGGTCAAACCCGGTCATTTAGAGGAAGTAAAAGTCGTAACAAGGTTTCCGTAGGTGA    1780
KF175513.1        AGTTGGTCAAACCCGGTCCCTTAA-GAAGGT-----------------------------    1726
                  **      *      **

KX950801.1        ------------------- 1273
MK371712.1        ------------------- 1698
MK371714.1        ------------------- 1689
XR_002735719.1    ACCTGCGGAAGGATCATTA 1799
KF175513.1        ------------------- 1726
```

S. 4 Multiple-sequence alignment of the sequences of amplified targeted ITS region
